# Whole body vibration, an alternative for exercise to improve recovery from surgery?

**DOI:** 10.1016/j.bbih.2022.100521

**Published:** 2022-09-24

**Authors:** Tamas Oroszi, Klaske Oberman, Csaba Nyakas, Barbara van Leeuwen, Eddy A. van der Zee, Sietse F. de Boer, Regien G. Schoemaker

**Affiliations:** a Department of Neurobiology, Groningen Institute for Evolutionary Life Sciences (GELIFES), University of Groningen, the Netherlands; b Research Center for Molecular Exercise Science, Hungarian University of Sports Science, Budapest, Hungary; c Behavioral Physiology Research Laboratory, Health Science Faculty, Semmelweis University, Budapest, Hungary; d Department of Surgery, University Medical Center Groningen, the Netherlands; e Department of Cardiology, University Medical Center Groningen, the Netherlands

**Keywords:** Whole body vibration, Exercise, Postoperative cognitive dysfunction, Neuroinflammation, Neurogenesis, Hemodynamics

## Abstract

Although exercise is usually associated with beneficial effects on physical and mental health, patients recovering from surgery may be hampered to perform active exercise. Whole body vibration (WBV) is suggested a passive alternative for physical training. Aim of the present study was to explore the therapeutic potential of WBV compared to physical exercise during early post-surgery recovery.

Male three months old Wistar rats underwent major abdominal surgery. Starting the day after surgery, rats were subjected to either daily WBV or exercise (treadmill running) for 15 consecutive days. Control rats underwent pseudo treatment. During the first week after surgery, effects of interventions were obtained from continuous recording of hemodynamic parameters, body temperature and activity (via an implanted transducer). Behavioral tests were performed during the second post-surgical week to evaluate anxiety-like behavior, short and long-term memory functions, cognitive flexibility and motor performance. Animals were sacrificed 15 days after surgery and brain tissue was collected for analysis of hippocampal neuroinflammation and neurogenesis.

Surgery significantly impacted all parameters measured during the first post-surgery week, irrespective of the type of surgery. Effect on cognitive performance was limited to cognitive flexibility; both WBV and exercise prevented the surgery-induced decline. Exercise, but not WBV increased anxiety-like behavior and grip strength. WBV as well as exercise prevented the surgery-induced declined neurogenesis, but surgery-associated hippocampal neuroinflammation was not affected.

Our results indicated that active exercise and WBV share similar therapeutic potentials in the prevention of surgery induced decline in cognitive flexibility and hippocampal neurogenesis. In contrast to exercise, WBV did not increase anxiety-like behavior. Since neither intervention affected hippocampal neuroinflammation, other mechanisms and/or brain areas may be involved in the behavioral effects. Taken together, we conclude that WBV may provide a relevant alternative to active exercise during the early stage of post-operative recovery.

## Highlights

• Both whole body vibration (WBV) and running exercise restored the reduced cognitive flexibility caused by surgery.
• WBV as well as active exercise prevented surgery-induced declined neurogenesis.
• Active exercise, but not WBV, induced anxiety-like behavior after surgery.
• Neither WBV nor active exercise affected surgery-induced neuroinflammation.
• Neither WBV nor active exercise influenced hemodynamic recovery from surgery.

## 1 Introduction

Physical activity has been widely acknowledged to improve general health and fitness (Warburton, 2006); and it is associated with improved muscle strength (Beaudart et al., 2017), bone mineral density (Benedetti et al., 2018) and oxygen uptake (Hawley et al., 2018). Another prominent effect of physical activity is to promote brain activity and preserve brain functions in humans (Kirk-Sanchez and McGough, 2013) and animals (van Praag, 2005) in health as well as in disease (Ma et al., 2017). These effects may be attributed to the anti-inflammatory actions of exercise (Seo et al., 2019; Mee-inta and Zhao, 2019). Therefore, physical activity would be a rational route to improve brain functions in various pathological and non-pathological conditions associated with neuroinflammation. However, for various reasons not all patients are capable and/or motivated to perform physical exercise.

One of the conditions that could hamper patients from performing exercise is recovery from major surgery. More than 200 million surgical procedures are performed worldwide in the last years and it is estimated that 50% of older individuals (>60) has had one surgical procedure during their life (Dodds et al., 2017). The advanced surgical and anesthetic techniques, combined with enhanced longevity, come with an increased risk of surgery associated complications (Krenk and Rasmussen, 2011). One of these complications is postoperative cognitive dysfunctions (POCD), a complex cluster of delayed recovery of cognitive functions and development of depression (Dodds et al., 2017). POCD is associated with accumulation of cytokines and microglia activation, and impairment of various neurotransmitters and neurotrophic pathways (Krenk et al., 2010). However, although physical activity could play a beneficial role in post-surgical rehabilitation, patients may not be capable to perform physical exercise with sufficient intensity and volume early after surgery.

Passive exercise stimulation by special vibratory platforms, known as whole body vibration (WBV) might provide an alternative. In general, WBV seems to mimic the beneficial effects of active exercise, including increased muscle strength (Annino et al., 2007, 2017), bone density (Slatkovska et al., 2010) and aerobic fitness (Zhang et al., 2014). Accordingly, WBV has been considered a feasible alternative for exercise to improve brain functions (Fuermaier et al., 2014a, 2014b). Moreover, WBV can be applied as passive exercise, since the patient is only exposed to vibration and do not perform active movements, hence requiring less energy expenditure and physical exertion. The potential of passive WBV as early postoperative therapy was successfully demonstrated in terms of improved neuromuscular control (Fu et al., 2013), increased knee extensor strength and decreased calf swelling (Hsiao et al., 2019). In addition, WBV can be passively applied to patients that are bed bound or ICU (intensive care unit) bound (Sañudo et al., 2020). These findings suggest that WBV as passive exercise stimulation might offer a well controllable exercise strategy with relative low intensity for populations with limited physical and/or mental capabilities.

Although POCD has become more acknowledged now, the underlying pathophysiology is far from understood, and consequently the rational for therapy. Preclinical studies may help to gain more insight. Therefore, during the last decades, we developed and tested an animal model for POCD (Hovens et al., 2014a). In this model we showed temporal postoperative cognitive dysfunction and neuroinflammation in young healthy rats (Hovens et al., 2014a), which was exaggerated in case of known risk factors in patients, such as advanced age (Hovens et al., 2013), previous infectious disease (Hovens et al., 2015), or type of surgery (Hovens et al., 2012). Moreover, pretreatment with ibuprofen, as standard anti-inflammatory therapy, was able to prevent cognitive decline in this model (Oberman et al., 2021). These results are in line with literature (Wan et al., 2007; Cibelli et al., 2010).

Preclinical studies have shown that WBV may stimulate different brain regions and promotes the synthesis of neurotransmitters (Ariizumi and Okada, 1985; Zhao et al., 2014; Heesterbeek et al., 2017) and neurotrophic factors (Huang et al., 2018; Raval et al., 2018), alleviates (neuro)inflammation (Peng et al., 2021; Chen et al., 2022; Oroszi et al., 2022a); and improves cognitive functions (Keijser et al., 2017; Boerema et al., 2018). Additionally, WBV may improve wound healing (Weinheimer-Haus et al., 2014; Wano et al., 2021). Taken together, we hypothesize that WBV could provide a safe and low-cost alternative for active exercise during the early phase of postoperative recovery.

The aim of the present study was to explore WBV as an alternative for active physical exercise in rats recovering from surgery, by assessing peripheral physiological parameters, neuroinflammation and neurogenesis in the brain, motor performance, and mood and cognitive behaviors.

## 2 Material and methods

### 2.1 Animals and housing

Male 3 months old Wistar rats (Janvier, Saint-Isle, France) were kept under conventional conditions in our laboratory at a reversed 12-12 h light-dark cycle (light on at 21:00), at constant temperature of 22 ± 2° Celsius and humidity of 50 ± 10%. Rats were group housed in standard home cages until the moment of surgery (3–4 animals per cage). After the surgical procedure animals were housed individually (45x30x50) to avoid damage to the implanted catheter. Water and food (standard rodent chow: RMHB/2180, Arie Block BV, Woerden, NL) were available ad libitum. All procedures were approved by the national Competent Authority (CCD) and the local ethical animal welfare committee (IvD) of the University of Groningen, The Netherlands.

### 2.2 Experimental groups and study design

Animals were randomly assigned to eight experimental groups:

**Non-Surgery (n=14)**

C1. Non-Surgery – home cage control (n = 7)

C2. Non-Surgery – pseudo WBV/Running (n = 7)

**Pseudo Exercise – Surgery (n=16)**

S1. Abdominal Surgery – pseudo WBV/Running (n = 8)

S2. Telemetry Surgery – pseudo WBV/Running (n = 8)

**WBV - Surgery (n=16)**

V1. Abdominal Surgery – WBV (n = 8)

V2. Telemetry Surgery – WBV (n = 8)

**Running Exercise - Surgery (n=15)**

R1. Abdominal Surgery – Running (n = 8)

R2. Telemetry surgery – Running (n = 7)

**Fig. 1:**
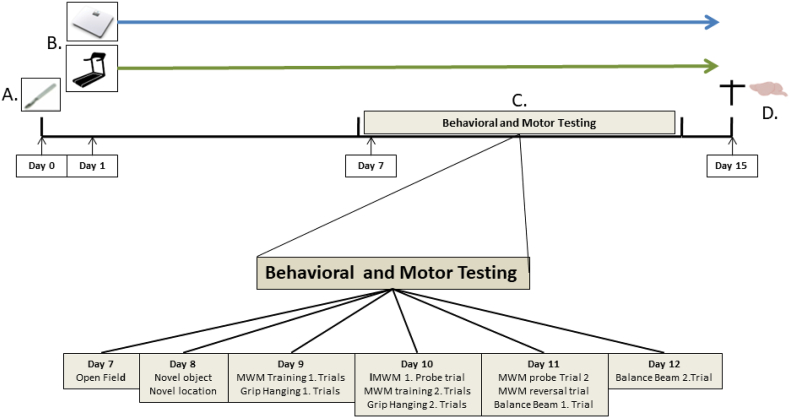
General Experimental Design: (A) Animals underwent a standard abdominal surgery or telemetry probe surgery to obtain measurements of baseline physiological parameters (day 0). (B) From day 1 to day 14, active exercise (treadmill running (running exercise – surgery)) or passive exercise (WBV – (WBV – surgery)) or pseudo intervention (pseudo exercise – surgery) was performed. (C) From day 7 to day 13 behavioral tests were conducted including open field, novel object and novel location recognition, Morris water maze (MWM), grip hanging and balance beam tests. (D) Animals were terminated and brain tissue was collected at day 15.

The experimental protocol is illustrated in Fig. 1. Rats received two days of familiarization (week −1) to the training environment. For that, on separated days all rats were placed once on the vibration platform (for 10 min) without vibration and once on the turned-off treadmill (for 30 min). Subsequently, rats underwent abdominal surgery to be able to compare outcomes with previous studies in this model (Hovens et al., 2014a) or telemetry probe implantation to obtain measurements of circadian rhythm, hemodynamics and body temperature (Sgoifo et al., 1996). Since three animals died shortly after telemetry surgery (two animals from telemetry/running group and one animal from telemetry/WBV group), to balance the experimental groups, two random chosen rats, initially aimed to serve as non-surgery controls (one home cage and one pseudo exercise), were transferred to the telemetry/running and telemetry/WBV groups. Essential aspects of the two surgical procedures were regarded similar, as those include major abdominal intervention with temporal arterial occlusion and placement of a permanent intravascular catheter. After surgery, rats were housed individually. Non-surgery rats served as control. These non-surgery control rats were also transported to the surgery room, left there for the time of surgery as comparable to the rats that underwent surgery, and were individually housed subsequently. All rats were returned to the housing facility at day 0. Rats with a telemetric probe were placed in their home cage on a receiver to obtain continuous recordings. One day after surgery, active (running) exercise or passive WBV was started and continued until sacrifice. From day 7–13 after surgery, rats were subjected to behavioral testing regarding mood, cognition and motor performance. Animals were sacrificed at day 15. Brain tissue was collected for immunohistochemical analyses of (neuro)inflammation and neurogenesis.

### 2.3 Surgical procedures

#### 2.3.1 Abdominal surgery and jugular vein catheter implantation

Under general anesthesia (2.5% isoflurane in 0.9 L oxygen/air) and buprenorphine analgesia (0.01 mg/kg s.c.), rats underwent abdominal surgery combined with jugular vein catheter implantation, as described in detail in our previous studies (Hovens et al., 2014a). Briefly, the abdominal cavity was opened along the linea alba to exteriorize the intestines and clamp the upper mesenteric artery for 30 min. During these 30 min, a silicon catheter (0.95 mm of outside diameter) was implanted into the right jugular vein, while the other end of the catheter was fixed on the top of the skull with dental cement. After this procedure, the intestines were placed back into the abdomen cavity and the muscles layer and skin were closed separately. This abdominal surgery procedure had been developed to mimic the effects of major abdominal surgery in humans.

#### 2.3.2 Transmitter implantation surgery

Under anesthesia and analgesia (see above), rats underwent a radio telemetry transmitter implantation, as described in detail elsewhere (Sgoifo et al., 1996). Briefly, the abdominal cavity was opened through incision of the linea alba. Intestines were kept aside with saline soaked gauze. The abdominal aorta was freed from surrounding tissue and clamped. The sensor of the probe (Data Science International, HD-S10) was inserted into the abdominal aorta. The transmitter was placed into the abdominal cavity and sutured to the inner abdominal wall for fixation. In contrast to the standard abdominal surgery (2.4.1), the impact on postoperative brain and behavioral parameters of the surgical procedure for transmitter implantation has not yet been studied.

### 2.4 Interventions

#### 2.4.1 Whole body vibration (WBV)

For WBV, a low intensity vibration device (MarodyneLiV – Low Intensity Vibration; BTT Health GmbH; Germany) was used to ensure constant vibration for rats of about 300 g (Marodyne plate is depicted in Fig. 1). This platform offers constant synchronous vertical vibration exposure with a sinusoidal nature at the frequency of 30 Hz and the amplitude of 50– 200 μm for a subject weighted in the range of 20–120 kg. The frequency range of 20–40 Hz and low amplitude (less than 0.1 mm) has been generally used to improve osseous and muscle parameters in rodents (Prisby et al., 2008). Further, this type of Marodyne platform with the same frequency and amplitude parameters and 10 min daily sessions was already successfully used in our previous experiments to ameliorate the age-related decline in spatial memory, neuroinflammation and motor performance in Wistar rats (Oroszi et al., 2022a).

Since the subject to vibration needs to be in the weight range of 20–120 kg, it was essential to adjust the platform for rats by placing a metal plate on the top of the vibration platform. Rats were placed in empty individual cages (45x30x50) on the platform (same shape and size as the individual home cage, but without bedding) (i.e.: on the top of the metal plate). Animals started with one 10 min session on the first post-surgical day (at 16 p.m.). From the second postsurgical day, animals were exposed to twice a day 10 min WBV with a 6 h interval, for 6 consecutive days (at 10 p.m. and at 16 p.m.). This was conducted in a separated experimental room under light conditions, but with the same climate parameters as the housing room. To avoid interference with cognitive and motor testing, during the second week, daily sessions were reduced to one per day, and performed after the behavioral testing procedures (at 16 p.m.). Animals always had a 16 h long resting period between the last vibration session and the start of cognitive and motor testing. After the behavioral test week, animals returned to two sessions of WBV per day until sacrifice.

Animals received prior habituation to the treatment procedure and environments without training exposure on week −1 before the surgery (i.e.: 1 day on the vibration platform and 1 day on the treadmill). It is important to note, that during the WBV treatment, the animals were not fixated in the cages and did not show signs of unprompted motor activity and stayed mainly in sitting or laying position during the 2 weeks of intervention. Further, animals did not show acute, short-term or long-term side effects of vibration. The parameters of vibration with the experimental setup were verified by additional measures by use of a 3D-accelerometer in the center (frequency: 29.6 Hz; peak-to-peak amplitude: x: 0, y: 0 z: 0.03 mm) and in the corners of cage (frequency: 29.6 Hz; peak-to-peak amplitude: x: 0, y: 0 z: 0.02 mm) used for the treatments. We adhered to the new reporting guidelines for WBV studies in animals (van Heuvelen et al., 2021).

#### 2.4.2 Running exercise

Running exercise was performed on a treadmill (Home-made, University of Groningen, The Netherlands). Animals started to train one day after the surgery and the training sessions were performed once a day (at 16 p.m.) for 15 consecutive days. During the first days (1.-3. days), the velocity was set low (5–6 m/min) and was gradually increased to reach the aimed speed of 18 m/min: ± 65% of the maximum oxygen uptake (Høydal et al., 2007). The duration of each training session was kept constant at 30 min. Rats were stimulated to run by paper barriers at the bottom of the treadmill to keep them on the treadmill. No electric shocks were used.

#### 2.4.3 Pseudo WBV/Running

Based on pilot studies, pseudo WBV/exercise rats received combined pseudo vibration and pseudo running treatment in order to serve as control for both interventions. The two pseudo interventions were rotated every day; one day twice 10 min (once 10 min during the testing phase) on the vibration plate without vibration – the other day 30 min on the turned-off treadmill.

### 2.5 General recovery parameters

#### 2.5.1 Body weight

Animals were weighted on day −3 and −5 before surgery. After surgery, bodyweights were obtained every day at the end of the light phase (between 8:00–8:30 a.m.). The change of the absolute body weight (weight lost compared to the baseline level of body weight before the surgery) and the maximum weight loss within the first 5 days were determined.

#### 2.5.2 Physiological parameters

Radio telemetry HD-S10 transmitters (Data Science International, USA) were implanted to continuously monitor heart rate (HR - beats/min), core temperature (CT - °C), blood pressure (BP - mmHg) and locomotors activity (LOC - counts/minute, cpm). Sampling rate was set at 1 min and all parameters were taken in 15 s intervals. Data were collected from the second day after surgery until day 6 after surgery to determine early post-operative recovery, and at day 14 for later effects. Values of HR, CT, BP and LOC were obtained by radiotelemetry HD-S10 transmitters and were assessed as 24 h means, and as 12–12 h separated dark and light cycles on each day. In addition, delta heart rate of the light/dark cycles was calculated to describe circadian rhythmicity.

### 2.6 Behavior

Behavioral changes, cognitive and motor performance were assessed during the second week of the protocol; sequence of tests is depicted in Fig. 1. Behavioral tests were performed during the dark phase (= active phase), excluding the first and last 2 h, in a separated experimental room under reduced light conditions, but with the same climate parameters as the housing room.

#### 2.6.1 Mood; open field test

An open field (OF) test (post-surgery day 7) was conducted to assess exploratory behavior and mood (Hovens et al., 2014a). For this, rats were placed in a square test box (100x100 × 40 cm), which was virtually divided into a center area (60 × 60 cm), 4 corner areas (20 × 20 cm) and the 4 side-wall areas (20 × 60 cm). Rats were allowed to explore this area for 5 min, while behavior was recorded. The total distance walked was determined by Ethovision (Noldus Information Technology, Wageningen, The Netherlands), while frequency and duration of rearing behavior (i.e.: when the rats raised on their hind limbs) were analyzed with the Eline software (University of Groningen, the Netherlands), as measures for exploration. Time spent in the corners and number of visits to the center were used as measure for anxious/depressive-like behavior; mood. Between the tests, the box was cleaned by 70% of ethanol solution and dry paper towel to avoid smell from the previous rat influencing the test.

#### 2.6.2 Cognition

##### 2.6.2.1 Short-term memory

After habituation to the test box (50x50 × 40 cm) on postoperative day 7, rats were subjected to short-term object- and location memory in the Novel Object Recognition (NOR) and Novel Location Recognition (NLR) tests (Hovens et al., 2014a), respectively, on postoperative day 8. The test series consisted of four phases of 3 min, separated by 1 min. The rats were remained in the box during the entire test series. Briefly, the rats were placed into the empty box for 3 min and habituated to the test environment. In the exploration phase, two identical objects (plastic or glass bottles) were placed into the box and the rats were allowed to explore these for 3 min. Then the objects were removed. In the NLR the same two objects were placed back, but one object in a different position, whereas in NOR one of the original objects was replaced by a novel object. The order of NLR and NOR phase was randomized. The size of the two objects was similar, but their shape was different. Between each phase, the objects were cleaned by 70% of ethanol solution and dry tissue. The test procedure was recorded for later analyses by Eline software (University of Groningen, the Netherlands). Preference was calculated as the ratio of time exploring the novel or relocated objects compared to the total object exploration time and used as outcome measurement of spatial and object memory. Rats that did not explore the objects or only explored only one of them were excluded from statistical analyses.

##### 2.6.2.2 Long-term learning and memory (Morris Water Maze)

The Morris water maze (MWM) test was conducted to assess long-term spatial learning, spatial memory, memory consolidation and cognitive flexibility (Hovens et al., 2014a). The test was performed using a round pool (diameter 140 cm) filled with water of 26 ± 1°Cand spatial cues on the wall. The pool was virtually divided into 4 quadrants. A platform (1 cm below the level of water; invisible for the rat) was placed in one quadrant called the target quadrant.

MWM test series started on the postoperative day 9 with 3 consecutive training sessions with 1.5 h between each session, and continued on day 10 and 11. Each training session consisted of 3 consecutive trials, in which the rat was placed with face towards the wall, randomly in each quadrant not containing the platform, and allowed to search for the platform during 1 min. The trial was stopped 10 s after the rat found the platform. If the rat was not able to find the platform within 1 min, it was gently guided towards the platform by the researcher and left there for 10 s. After finishing of the session (3 trials), the rats were toweled dry and returned to their home cage. The order of the rats was randomized, but preserved over the three sessions, providing the same time gap between the three sessions of each rat. The average escape latency of three trials per session was used to construct a learning curve.

The second day of the protocol started with the probe trial to assess early development of spatial memory. For that, the platform was removed from the pool. Rats were placed in the quadrant opposite to the previous target quadrant and allowed to explore the maze for 1 min. Percentage of the time spent in the target quadrant and the number of crossings over the previous position of the platform (platform crossings) were measured and analyzed during first 15 s and served as marker for early spatial memory. Pilot studies indicated that this premature memory test did not affect spatial learning. After this probe trial, animals performed another 3 training sessions to consolidate the platform location, as well it served as a baseline for the reversal training.

On the third day of the protocol a second probe trial was conducted to repeat the assessment of spatial memory following the same procedure as the first probe trial. After the probe trial, animals were tested for cognitive flexibility by reversal training, which consisted of three sessions (three trials per session), with the platform relocated in the opposite quadrant of the original target quadrant. Average escape latency of the three sessions were recorded and utilized as measure of cognitive flexibility. The difference between the latency in the last learning session and the first reversal learning session was regarded memory consolidation (Oberman et al., 2020).

During the three days of MWM, recordings were obtained and analyzed by automatic computerized image analyzing system (Ethovision, Noldus Information Technology, Wageningen, The Netherlands).

#### 2.6.3 Motor performance

##### 2.6.3.1 Balance beam

Balance beam test was conducted twice on postoperative day 11 and 12 to analyze the motor coordination (Oroszi et al., 2022b). A 150 cm long and 2 cm wide wooden slat was positioned horizontally at 1 m above the floor, and on one end connected to the home cage of the rat, as target. On the first day the rats were trained to walk across the suspended beam, at which the rats performed three trials (50 cm, 100 cm and one full test (150 cm)). After these 3 training trials, the rats performed 3 full test trials. On the second day, the rats performed also full 3 test trials. Video records were achieved about all trials and analyzed by visual observation. Average latency of the 3 best results to reach the home cage was used as measure for performance on the balance beam. In addition, animals who were unable and/or unwilling to perform the test procedure were excluded at the final statistical analysis.

##### 2.6.3.2 Grip strength test

Grip strength test was performed twice on postoperative day 9 and 10, included 3 trials per day (Oroszi et al., 2022b). Animals were held by their trunk and were guided to get grip on a suspended wood beam (2 mm in diameter, 35 cm in length, 50 cm above the surface of table) by their forepaws. Time until drop-off was recorded. The six trials of the two days were utilized for statistical analysis. During the three trials the animals were rotated to offer time for recovery between the trials.

### 2.7 Sacrifice

At day 15 after surgery, rats were anesthetized with pentobarbital (60 mg/kg, ip) and transcardially perfused with 4% of paraformaldehyde (PFA) containing 500 u/ml heparin. Whole brains were removed and immersion fixed with 4% PFA for 3 days. After post fixation brains were dehydrated for cryoprotection by 30% of sucrose solution followed with freezing by liquid nitrogen and stored at −80°C. Brains were cut into 25 μm thick sections. Free floating sections were stored in phosphate-buffered saline containing 0.2% sodium azide (PBSA) at 4 °C.

### 2.8 Effects on the brain

#### 2.8.1 Neuroinflammation; microglia activity

Neuroinflammation was obtained from microglia activity (Hovens et al., 2014a). To visualize microglia, sections were incubated for 3 days on 4 °C in cold room on shaker. Then sections were incubated with primary antibody (Wako, Neuss, Germany – Rabbit anti IBA-1) in 1:2500 dilution with 2% bovine serum albumin (BSA) and 0.01 Triton, followed by the incubation with 1:500 concentration of secondary antibody (Jackson, Wet Grove, USA – Goat Anti Rabbit).

Microglia activity was determined in the Prefrontal Cortex (PFC) and the CA1 and Hilus regions of the dorsal hippocampus. For that, photographs were taken from the sections at 200× magnification. By using of image analyze software (Image Pro Plus 6.0), microglia density, coverage, total cell size, cell body size and dendrites size were determined. Microglia activity was calculated as the cell body to total cell size ratio (Hovens et al., 2014b).

#### 2.8.2 Neurogenesis; DCX

To analyze newly formed neurons in the hippocampus, doublecortin (DCX) staining was used (Hovens et al., 2014a). Sections (3 sections per animal) were blocked for 1 h by 5% bovine serum albumin (BSA). Sections were incubated with primary antibody (Santa Cruz, Dallas, USA – Goat-anti DCX) in 1:1000 concentrations with 1% BSA, 0.1% Triton in 0.01 M PBS for 3 nights on 4 °C in cold room on shaker, followed by incubation of secondary antibody (Jackson, Wet Grove, USA – Rabbit-Anti Goat) in 1:500 dilution with 1% BSA, 0.1 Triton in 0.01 M PBS for one night.

Pictures were taken from the dorsal hippocampus with light microscope at 40× magnification. The area of DCX positive structures in the dentate gyrus (DG) was determined and corrected for the length of the DG by image analysis (Image Pro Plus 6.0.), as measure for neurogenesis.

### 2.9 Statistical analysis

Statistical analysis was performed by Statistica 13.2 software. Data are presented as group means ± standard error of the mean (SEM). Effects of pseudo treatment versus home cage control were analyzed by comparison of the 2 non-surgery control groups (C1 and C2) using an independent sample T-test. Similarly, the 2 types of surgery were compared, irrespective of intervention, using an independent sample T-test. In case of no statistically significant difference, data of groups with the same treatment were pooled.

Spatial learning and cognitive flexibility in MWM, and outcomes of the early telemetry measurements were assessed by using repeated measures ANOVA: between subject factors were “main groups” (1. Running exercise/Surgery; 2. WBV/Surgery; 3. Pseudo exercise/Surgery; 4. Non-surgery) or “telemetry groups” (1. Running exercise/Telemetry; 2. WBV/Telemetry; 3. Pseudo exercise/Telemetry). Within subject factors were “training trials” (6 levels, trial 1 to trial 6) or “reversal trials” (3 levels, trial 1 to trial 3) in the MWM; and were “day of measurements” (6 levels, day 1 to day 6) and “dark – light” (2 levels, dark and light cycle) in the early telemetry measurements.

All other outcome variables were compared by one-way ANOVA with the factor “main groups” (1. Running exercise/Surgery; 2. WBV/Surgery; 3. Pseudo exercise/Surgery; 4. Non-surgery) or “telemetry groups” (1. Running exercise/Telemetry; 2. WBV/Telemetry; 3. Pseudo exercise/Telemetry) followed by Tukey HSD post hoc analysis corrected for multiple comparison. In addition, in case of MWM probe trials, all groups were compared to the 25% of chance level by a one sample T-test.

For revealing potential relevant correlations between the behavioral outcomes (exploration, mood, cognition and motor performance) and the telemetry measures and brain related markers of this experiment a parametric Pearson correlation analysis was used.

Data which exceeded mean ± twice standard deviation of its group were considered as outlier (no more than 1 animal per group) and were omitted from analyses. Statistical significance was set at p < .05. Relevant tendencies (p < .1) were also presented. In addition, effect size was calculated by using Cohen's d to further investigate these effects and the following classification bechmarks were considered: small effect: 0.2 < d < 0.5; medium effect: 0.5 < d < 0.8; and large effect: d > 0.8.

## 3 Results

### 3.1 General

#### 3.1.1 Effects on body weight

On average rats weighed 369 ± 4 g before surgery, with no difference between the groups. From 64 rats (16 per group), 3 rats died during or shortly after surgery. None of the measured parameters differed significantly for the non-surgery home cage controls and non-surgery pseudo WBV/exercise control rats. Therefore, data from these non-surgery subgroups (C1 – non-surgery home cage control and C2 – non-surgery pseudo WBV/exercise) were pooled into one non-surgery control group.

**Fig. 2:**
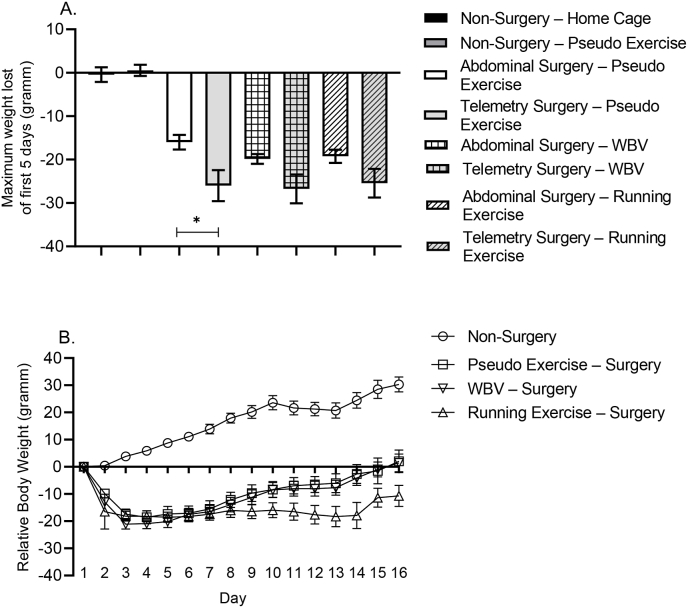
Effects of standard abdominal surgery and telemetry surgery on maximum weight loss within the first 5 days (panel A). Panel B: Body weight over the time course of the study in experimental groups pooled according to intervention. Data are depicted as mean ± SEM. (Non-Surgery n: 16 (7 home cage and 7 pseudo exercise); Pseudo exercise – surgery n: 16 (8 abdominal – 8 telemetry); WBV – surgery n: 16 (8 abdominal – 8 telemetry); Running exercise – surgery n: 15 (8 abdominal – 7 telemetry).

Maximum body weight loss during the first 5 postoperative days was calculated to compare the impact of both types of surgeries and the effects of intervention on body weight. Surgery rats showed significant body weight loss within the first 6 days after surgery, which was more pronounced in transmitter implanted rats than in abdominal surgery rats, but was not significantly affected by either intervention (Fig. 2A). Moreover, maximal weight loss after surgery appeared to be the only parameter that distinguished between the abdominal and transmitter surgery. Behavioral and brain data from the two types of surgery did not differ significantly; data were therefore pooled, resulting in 3 surgery groups. Taken together, data were presented as the following major groups: 1) non-surgery; 2) pseudo exercise – surgery; 3) WBV - surgery and 4) running exercise – surgery for further analyses.

Accordingly, Fig. 2B presents a time course of body weight during the whole study for the 4 pooled groups. Indeed, whereas non-surgery control rats continued gaining body weight, all surgery rats lost weight after surgery. During the second week, the behavioral testing week, body weight stabilized in all groups, though at the lowest values for the exercise rats. Pseudo exercise – surgery rats and WBV– surgery, but not running exercise – surgery rats, returned to their original weight at the end of the study.

#### 3.1.2 Running performance

Running performance was monitored from the 2nd day to 15th day of the intervention and standard abdominal surgery and telemetry surgery groups (R1 and R2) were compared. Average running distance was recorded during each training sessions and area under the curve (AUC) was calculated to determine the total amount of workload during the intervention. On average, running distance was 205 ± 14 m during the first training session, with no difference between the groups. However, over the entire study protocol the rats that underwent abdominal surgery ran more distance than the telemetry surgery rats (Supplementary Figs. 1A and B).

### 3.2 Physiological parameters

Early recovery was obtained from physiological parameters measured with the telemetric device during the first 6 days after surgery, while late effects were obtained from these parameters on day 14 after surgery.

#### 3.2.1 Early effects

**Fig. 3:**
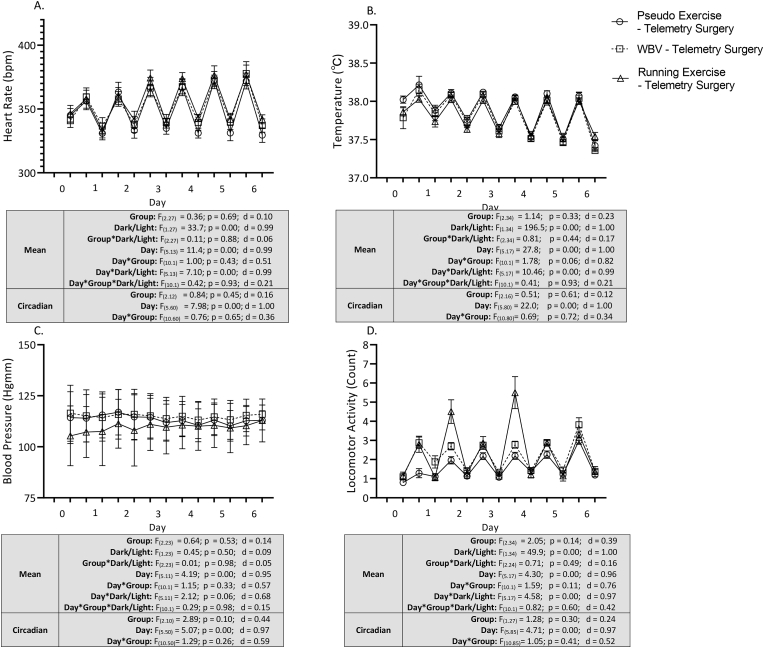
Early effects of telemetry surgery (pseudo exercise), and WBV and running exercise – telemetry surgery interventions on baseline physiological parameters including heart rate (panel A), core temperature (panel B), mean blood pressure (panel C) and on locomotor activity (panel D). Data are depicted as values during the dark phase (from day 1 to day 6) and during the light phase (from day 0 to day 6). Statistical outcome represents 24 h mean (dark and light phase) and circadian rhythmicity. Mean values as well as circadian rhythms of all parameters showed significant time course alterations during the experiment, without significant effects of interventions or interaction of time course and interventions. Data are depicted as mean ± SEM. (Heart Rate: pseudo exercise – surgery n = 6, WBV – surgery n = 7, running exercise – surgery n = 6; Temperature: pseudo exercise – surgery n = 7, WBV – surgery n = 8, running exercise – surgery n = 7; Blood Pressure: pseudo exercise – surgery n = 5, WBV – surgery n = 7, running exercise – surgery n = 5; Activity: pseudo exercise – surgery n = 7, WBV – surgery n = 8, running exercise – surgery n = 7).

For early effects, heart rate (HR), core temperature (CT), mean blood pressure (BP) and locomotor activity (LOC) were analyzed during the first 6 days after surgery, regarding time course (recovery), effects of intervention, effects of dark/light cycle and interaction between these parameters. Time course of the effects are shown in Fig. 3. Data are depicted as separated dark – light cycles during the first 6 days. However, statistical outcomes were characterized by time course of 24 h mean values (mean of dark and light cycle) and circadian rhythmicity (i.e.: delta values of dark and light cycle). Statistics are shown underneath the graphs.

Repeated measure ANOVA revealed a significant time course for all parameters including mean values and circadian rhythmicity. Significant effect of light/dark phase were detected in heart rate, core temperature and locomotor activity, but not in blood pressure. These effects were corroborated by large effect sizes (d > 9). In contrast, for neither parameter, effects of intervention or interaction of time course and intervention were observed.

#### 3.2.2 Late-term effects

For late effects, heart rate (HR), core temperature (CT), mean blood pressure (BP) and locomotor activity (LOC) on day 14 were analyzed. Similarly, to the early effects, data were analyzed as mean values and circadian rhythmicity. No significant differences were found in the late recovery between the experimental groups (Table 1).

**Table 1:** Late effects of telemetry surgery (pseudo - exercise), WBV and running exercise – telemetry surgery interventions on circadian rhythmicity including heart rate, core temperature, mean blood pressure and locomotor activity. Data are depicted mean heart rate and circadian rhythmicity. Data are depicted as mean ± SEM. (Heart Rate: pseudo exercise – surgery n = 6, WBV – surgery n = 8, running exercise – surgery n = 6; Temperature: pseudo exercise – surgery n = 6, WBV– surgery n = 8, running exercise – surgery n = 7; Blood Pressure: pseudo exercise – surgery n = 5, WBV – surgery n = 7, running exercise – surgery n = 5; Activity: pseudo exercise – surgery n = 7, WBV – surgery n = 8, running exercise – surgery n = 7).

		Heart Rate (bpm)	Temperature (˚C)	Blood Pressure (mmHg)	Activity (count)
Mean	Pseudo Exercise	344 ± 5	37.6 ± 0.0	107 ± 3	2.26 ± 0.11
	WBV	351 ± 7	37.6 ± 0.0	114 ± 3	2.61 ± 0.12
	Running Exercise	345 ± 5	37.5 ± 0.0	110 ± 5	2.24 ± 0.19
Circadian	Pseudo Exercise	43 ± 4	0.6 ± 0.1	0 ± 0	1.78 ± 0.25
	WBV	25 ± 7	0.3 ± 0.1	1 ± 0	1.50 ± 0.40
	Running Exercise	34 ± 3	0.5 ± 0.0	0 ± 0	1.40 ± 0.08

### 3.3 Effects on behavior

#### 3.3.1 Exploration and mood

**Fig. 4:**
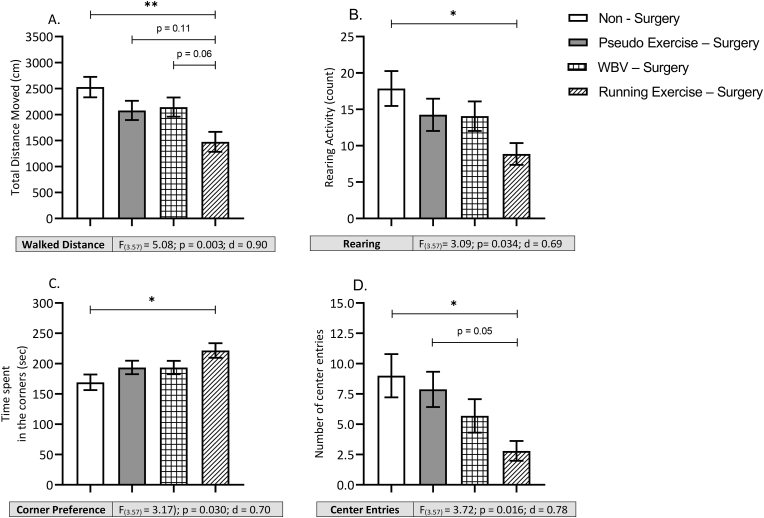
Effects of surgery (pseudo exercise), and WBV and running exercise interventions after surgery on exploratory and anxiety-related behavior in open field test. The total walking distance (panel A), in the number of rearings (panel B) and time spent in the corner (panel C) and the number of center entries (panel D). Data are depicted as mean ± SEM. *: P < .05. **P < .01. ***P < .001 (non – surgery n = 14, pseudo exercise – surgery n = 16, WBV – surgery n = 16, running exercise – surgery n = 15).

The open field (OF) test was used to assess parameters of exploration and mood. Data are summarized in Fig. 4. Exploratory behavior, obtained from distance moved and number of rearings, was significantly affected only in the running exercise – surgery group. Rats that performed running exercise after surgery walked less distance (Fig. 4A) compared to pseudo exercise - surgery rats and showed less rearing activity than non-surgery control rats (Fig. 4B); effects that were not observed in rats with WBV. Effects on mood were obtained from spatial preference of the rats, time spent in the corners (Fig. 4C) and number of visits to the center (Fig. 4D). Rats that exercised after surgery, but not rats that received WBV, spent significantly more time in the safe corner areas compared to non-surgery control rats and paid less visits to the center area compared to pseudo exercise - surgery control rats. In addition, effect size calculations revealed that effects were medium – large on exploratory behavior (rearing d = 0.69 and walking d = 0.90) and medium on mood (time in corner and center entries d = 0.70 and 0.78).

Assessment of correlations between parameters of behavior versus early (day 1 – day 6) and late (day 14) telemetry values revealed significant correlations between number of center entries (r = −0.50, p = .02) and rearing activity (r = −0.51, p = .02), and early heart rate values. Whereas, time spent in the corner (r = 0.41, p = .06) also showed a strong tendency of positive correlation to the short-term heart rate values. Outcomes of exploration and mood in the OF test did not show correlation to the long-term heart rate values.

#### 3.3.2 Cognition

##### 3.3.2.1 Short-term memory

In the exploration phase of the NLR and NOR tests no significant differences were observed in the preference time nor in the frequency of objects exploration, indicating no side preference or differences in fear for novelty. Animals who did not explore both objects and/or the familiar and replaced/novel objects in the NLR or NOR were excluded at the final statistical analysis (NLR: 3 animals from each surgery groups (pseudo exercise, WBV and running exercise) and 2 animals from the non-surgery control group; NOR: 2 animals from running exercise – surgery and WBV – surgery groups and 4 animals from the pseudo exercise – surgery group). Results for short-term memory in the NOR and NLR are presented in Table 2. Neither significant differences nor medium or large effects were observed between groups regarding outcome of these short-term memory tests.

**Table 2:** Effects of surgery (pseudo exercise), WBV and running exercise interventions on the preference time (%) for the novel location (NLR) and novel object recognition (NOR). Data are depicted as mean and ±SEM. (NLR - NOR: non – surgery n = 12–13, pseudo exercise – surgery n = 13–12, WBV – surgery n = 14–14, running exercise – surgery n = 12–14).

	Non - Surgery	Pseudo exercise - Surgery	WBV - Surgery	Running exercise - surgery
NLR	65.1 ± 6.2	68.2 ± 4.2	65.0 ± 4.6	75.6 ± 6.4
NOR	58.7 ± 7.6	71.5 ± 6.0	66.8 ± 3.7	71.3 ± 7.5

Long term learning and memory (Morris Water Maze).

**Fig. 5:**
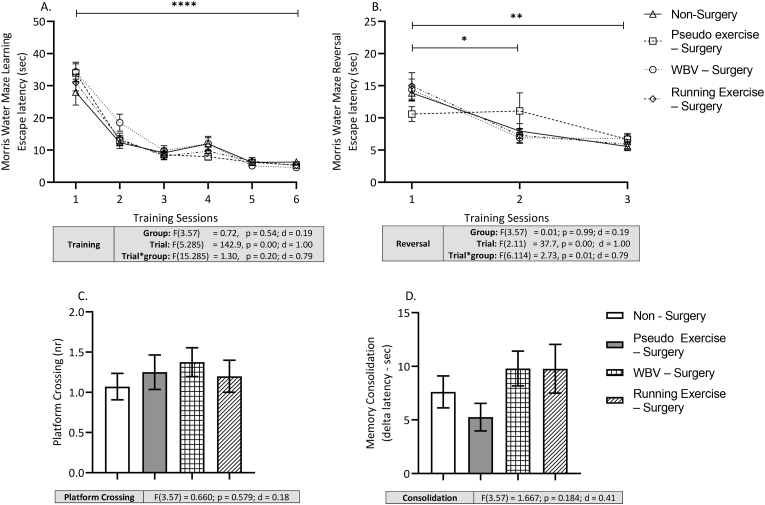
Effects of surgery (pseudo exercise), and WBV and running exercise interventions after surgery on long-term learning and memory in Morris water maze (MWM) test; learning curves of the 6 training sessions (day 1 and day 2) (Panel A); learning curves of 3 reversal trials (day 3) (Panel B). Platform crossing in the MWM second probe trial (day 3) (Panel C). Memory consolidation (last training sessions – first reversal) (Panel D). Data are depicted as mean ± SEM. Asterisks indicate: *P < .05. **P < .01. ***P < .001. (non – surgery n = 14, pseudo exercise – surgery n = 16, WBV – surgery n = 16, running exercise – surgery n = 15).

In the Morris Water maze all groups showed a significant learning curve (p < .001) with large effect size (d = 1.00), without differences between groups, indicating that all rats were able to learn the position of the platform (Fig. 5A). In the time spent in the target quadrant of the first and second MWM probe trial no significant difference between the experimental groups were observed. In addition, all groups performed significantly different from chance level of 25%, both at the first and second probe trials, indicating that all rats were equally able to learn and remember the position of the platform. In addition, number of platform crossing did not show differences between the groups during the probe trials (Fig. 5D).

However, in the MWM reversal training, in contrast to the non-surgery rats, the pseudo exercise - surgery rats did not show a learning curve anymore (Fig. 5B) (Trial x group p = .01). Moreover, this loss was completely reversed by both WBV and running exercise after surgery. This was corroborated with large effect size (d = 0.85). This finding may be reflected in memory consolidation, although not statistically significant and small effect size was observed (d = 0.41). Memory consolidation was substantially lower in pseudo exercise – surgery group compared to non-surgery rats, which was completely restored after WBV and running. (Fig. 5D).

### 3.4 Motor performance

**Fig. 6:**
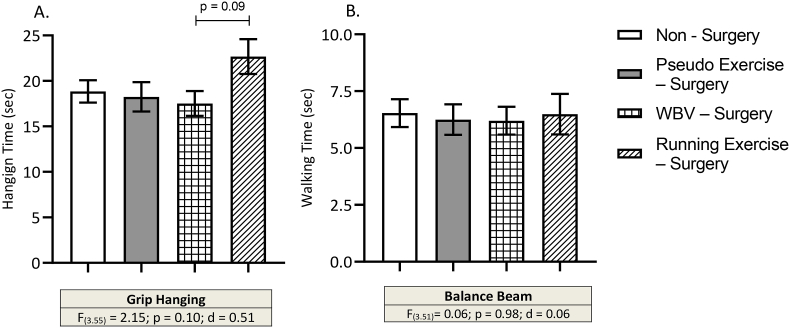
Effects of surgery (pseudo exercise), and WBV and running exercise interventions after surgery on muscle strength in grip hanging test (Panel A) and motor coordination in balance beam test (Panel B). Data are depicted as mean ± SEM. (Grip hanging/balance beam: non – surgery n = 13–13, pseudo exercise – surgery n = 16–16, WBV – surgery n = 16–16, running exercise – surgery n = 14–10).

Motor performance was obtained from the grip hanging test and the balance beam test. Surgery did not influence the outcomes of motor parameters. One-way ANOVA showed a tendency (p = .10) of increased grip hanging time with medium effect size (d = 0.51). Post-hoc analysis revealed that running exercise after surgery increased grip hanging time compared to WBV – surgery group (p = .09) (Fig. 6A). Balance beam performance was not significantly different between the experimental groups (Fig. 6B). Six animals were unable to perform the balance beam test and were excluded at the final statistical analysis. (5 animals from the running exercise – surgery group; 1 animal from the non-surgery control group).

In addition, assessment of correlations between the outcome of grip hanging test versus the outcomes of test found that grip strength was negatively correlated to the walking distance (r = −0.31; p = .01) and rearing activity (r = −0.31; p = .01).

### 3.5 Effects on brain

#### 3.5.1 Neuroinflammation

**Fig. 7:**
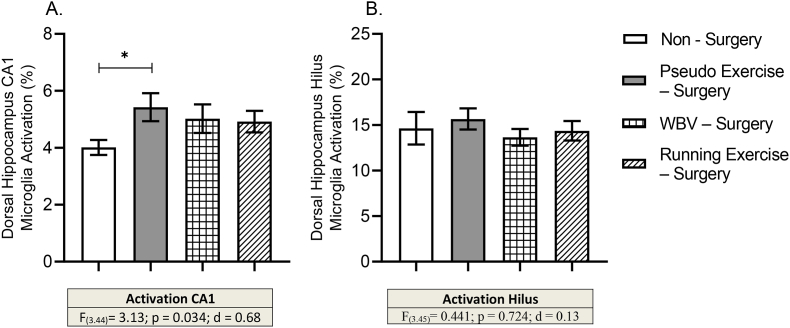
Effects of surgery (pseudo exercise), and WBV and running exercise interventions after surgery on microglia activity in subregions of dorsal hippocampus including CA1 (panel A) and Hilus regions (panel B). Data are depicted as mean ± SEM. * indicates: P < .05. (Non – surgery n = 10, pseudo exercise – surgery n = 13, WBV – surgery n = 14, running exercise – surgery n = 11).

Microglia activity was calculated for the PFC and the subregions of the dorsal hippocampus, including CA1 and Hilus regions. No significant differences were observed in any of the morphological parameters of microglia in the PFC between the experimental groups, including the calculated activity (data in supplement). However, while a tendency for correlation of PFC microglia activity with memory consolidation was observed (r = −0.243; p = .079), this was reflected in a significant negative correlation between cell body size and memory consolidation (r = −0.311; p = .023). One-way ANOVA revealed significant effect with medium effect size on microglia activation in the CA1 region (p = .03; d = 0.68). Additional post-hoc test showed that pseudo exercise – surgery group showed significantly higher degree of microglia activity compared to the non-surgery control group (Fig. 7A). Microglia activation was not significantly altered in the Hilus subregions. Furthermore, underlying morphological parameters in the CA1 and Hilus subregions did not show significant changes (Table 3).

**Table 3:** Effects of surgery (pseudo exercise), and WBV and running exercise interventions after surgery on microglia coverage, cell body size, dendrites size, total cell size and microglia density, measured in pixels, in the CA1 and Hilus subregions of hippocampus. Values of coverage and microglia per AOI were multiply with 1000 to improve readability. Data are depicted as mean and as mean ± SEM. (non – surgery n = 9–10, pseudo exercise – surgery n = 13–14, WBV – surgery n = 14–15, running exercise – surgery n = 11–12).

Group	Region	Coverage	Cell Body Area	Dendrites Area	Total Cell Size	Microglia per AOI
Non - Surgery	CA1	102 ± 10	439 ± 13	11160 ± 684	11599 ± 686	13 ± 1
	Hilus	147 ± 7	404 ± 17	2531 ± 305	2933 ± 312	34 ± 3
Pseudo Exercise – Surgery	CA1	139 ± 5	471 ± 23	8897 ± 733	9379 ± 735	15 ± 1
	Hilus	97 ± 6	375 ± 13	2289 ± 177	2664 ± 179	39 ± 3
WBV – Surgery	CA1	140 ± 6	440 ± 18	8969 ± 637	9415 ± 627	15 ± 1
	Hilus	115 ± 3	576 ± 17	2876 ± 250	3283 ± 261	38 ± 3
Running Exercise – Surgery	CA1	129 ± 6	447 ± 19	9296 ± 592	9743 ± 593	14 ± 0
	Hilus	107 ± 4	379 ± 13	2569 ± 242	2752 ± 163	40 ± 2

A significant negative correlation was also found for microglia activity in the CA1 region and to late heart rate (r = −0.63, p = .00) and locomotor activity values (r = −0.50, p = .03). In addition, microglia activity in the CA1 region also showed positive correlation with OF rearing activity (r = 0.31, p = .03).

#### 3.5.2 Neurogenesis

**Fig. 8:**
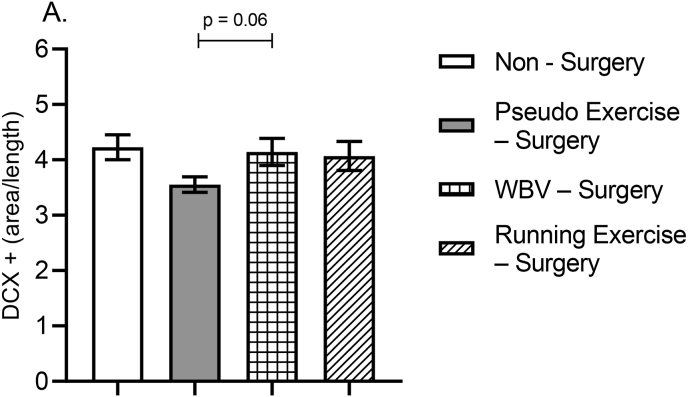
Effects of surgery (pseudo), and WBV and running exercise interventions after surgery on neurogenesis (DCX positive cells) in the dentate gyrus. Data are depicted as mean ± SEM. (non – surgery n = 14, pseudo exercise – surgery n = 15, WBV – surgery n = 16, running exercise – surgery n = 15).

One-way ANOVA showed a strong tendency (p = .08) with medium effect size (d = 0.56) in the coverage of DCX positive cells (Fig. 8A). Additional post-hoc analysis revealed that the WBV – surgery group had ameliorated level of DCX positive cells compared to the pseudo exercise - surgery control group (p = .06). In addition, a similar trend of increased level of DCX positive cells were observed in running exercise – surgery and non-surgery groups compared to the pseudo exercise – surgery control group.

Assessment of correlation between DCX positive cells and locomotor activity showed that DCX positive cells positively correlated to both early (r = 0.57, p = .00) and late values (r = 0.67, p = .00) of mean locomotor activity.

### 3.6 Correlations

Since behavioral outcome would be regarded the most important parameter of postsurgery recovery, correlation analyses was performed on behavior and early and late recovery parameters as well as late results of brain analyses (Table 4). Data revealed that exploration (rearings) was negatively correlated to early heart rate and positive to microglia activity in the CA1 at sacrifice. Mood was also correlated to early heart rate. However, short-term memory performance showed no correlation with early recovery parameters, but appeared positively correlated to microglia activity in the hippocampal CA1, and to neurogenesis. For motor performance no correlation with other parameters were observed.

**Table 4:** Notable correlations in behavioral tests compared the telemetry measure and molecular markers. Outcomes of exploration (rearing and distance moved), mood (corner time and center entries), cognition (novel location and object recognition) and motor performance (balance beam and grip hanging) were compared to early and late recovery of heart rate and body temperature, as well as to microglia activation (CA1, Hilus and PFC) and neurogenesis. Data are depicted as r and p values. Bold indicates significant correlation (P < .05).

		Heart Rate		Temperature		Microglia Activation			Neurogenesis
		early	late	early	late	CA1	Hilus	PFC	DCX+
Exploration	Rearing	**r=-.5121** **p=.021**	r = −.3926 p = .087	r = −.2675 p = .229	r = −.2876 p = .206	**r=.3140** **p=.030**	r = −.0081 p = .956	r = .0052 p = .971	r = .1517 p = .256
	Distance Moved	r = −.2744 p = .242	r = −.1646 p = .488	r = −.2026 p = .366	r = −.1428 p = .537	r = .2400 p = .100	r = −.1588 p = .276	r = .0166 p = .906	r = .0940 p = .483
Mood	Corner Time	r = .4184 p = .066	r = .2877 p = .219	r = .1884 p = .401	r = .2083 p = .365	r = −.2802 p = .054	r = .1534 p = .293	r = .0564 p = .689	r = −.1525 p = .253
	Center Entries	**r=-.5039** **p=.023**	r = −.3090 p = .185	r = .1992 p = .374	r = .0788 p = .734	r = −.2802 p = .554	r = .1534 p = .127	r = .0597 p = .671	r = −.1525 p = .665
Cognition	Novel Object Recognition	r = .3110 p = .259	r = .1896 p = .499	r = .1438 p = .595	r = −.0037 p = .989	**r=.3296** **p=.038**	r = −.0173 p = .914	r = −.2043 p = .168	r = −.1606 p = .260
	Novel Location Recognition	r = .0064 p = .981	r = −.2170 P = .403	r = −.1153 p = .649	r = −.1975 p = .432	r = −.0595 p = .715	r = −.2879 p = .068	r = −.0606 p = .693	**r=-.2984** **p=.039**
Motor Performance	Balance Beam	r = −.2312 p = .372	r = −.4637 p = .061	r = .2500 p = .302	r = −.0875 p = .730	r = .0657 p = .676	r = .0064 p = .967	r = −.2332 p = .093	r = −.2362 p = .089
	Grip Hanging	r = .2436 p = .301	r = .3780 p = .100	r = .1646 p = .464	r = −.0224 p = .923	**r=-.3123** **p=.033**	r = .0058 p = .969	r = −.0271 p = .856	r = .1274 p = .349

## 4 Discussion

### 4.1 General

The aim of the current study was to explore the therapeutic potential of WBV as alternative for active exercise training during early recovery from surgery. Heart rate, blood pressure, body temperature and spontaneous locomotor activity were affected by surgery but normalized in 5–6 days with no differences between the intervention groups. Running exercise, but not WBV, increased anxiety-like behavior. Neither short-term nor long-term learning and memory were affected by both interventions. However, both active running and passive WBV improved cognitive flexibility. Running exercise, but not WBV, increased muscle strength. Moreover, both interventions counteracted the declined level of hippocampal neurogenesis, while leaving the surgery-induced neuroinflammation unaffected.

Taken together, our data demonstrated that running exercise and passive WBV during post-surgical recovery share beneficial effects on neurogenesis and aspects of cognitive functions. However, the effects of WBV differ from those of exercise by the lack of anxiogenic effects and absence of enhanced muscle strength. As from the many parameters we obtained, some positive, but no negative side effects were observed after WBV. These results may indicate that WBV could provide a relevant alternative for exercise in early recovery after surgery, warranting explorative studies in human target groups.

### 4.2 Effects on early recovery

Body weight loss (approximately 5%) after abdominal surgery seemed in line with our previous work (Hovens et al., 2014a). Telemetry surgery caused more body weight loss than standard abdominal surgery, and may relate to the size of the implanted sensor (Helwig et al., 2012). Results indicated that physiological parameters, as well as locomotor activity were significantly affected mainly on the first two days after surgery, and were restored to normal values within 5–6 days post-surgery. The appropriate post-surgical recovery period of rats after telemetry surgery for reliable data collection in pharmacological studies was indicated to be one week, which would be in line with our findings (Greene et al., 2007).

The time course and progression of recovery of physiological parameters were not affected by running exercise or WBV intervention. To the best of our knowledge, there are no previous studies regarding the effects of running exercise or WBV on physiological parameters during the early stage of post-operative recovery in rodents. Alternatively, effects of long-term WBV stimulation on hemodynamics have been investigated in clinical studies, indicating decreased resting blood pressure (Figueroa et al., 2012; Beijer et al., 2013), supported by muscle tissue microcirculation and oxygenation (Rittweger et al., 2010; Beijer et al., 2015). However, in general, the influence of WBV on various cardiovascular parameters seemed to be moderate or even negligible. Hence, from a hemodynamic point of view, WBV could be considered as a save treatment strategy for individuals with limited active exercise capacity due to recent surgery. Moreover, our pilot study, measuring hemodynamics during WBV, also showed no effects (data not shown), strengthening that WBV can be a safe treatment strategy.

In contrast, the late cardiovascular adaptations, including resting sinus bradycardia and/or thermoregulation induced by active exercise training have been widely acknowledge in preclinical studies in rodents (Perrino et al., 2011; Abreu et al., 2016). However, adaptation is stimulation dependent (Seo et al., 2014; Abreu et al., 2016) and studies with short-term interventions (two weeks of exercise) including assessment of cardiovascular parameters in conscious rats are limited. The absence of cardiovascular adaptations in the present study may then be attributed to the combination of (passive and active) duration of exercise training and recovery from surgery.

### 4.3 Effects on behavior

The OF test was used to evaluated effects on exploratory behavior and anxiety-like behavior. Our findings demonstrated that OF behavior was not affected by surgery in the second post-operative week, which is in general agreement with our previous study at this time frame after surgery (Hovens et al., 2014a). However, running exercise seemed to induce anxiety-like behavior. We recently reported similar findings after exercise intervention (treadmill running) in isoproterenol-induced cardiac infarction rats (Tóth et al., 2021). It should be noted that in most studies various exerice interventions, including voluntary (Fulk et al., 2004; Liu et al., 2012; Uysal et al., 2018; Caliskan et al., 2019) and involuntary forms (Binder et al., 2004; Duman et al., 2008; Salam et al., 2009), have been generally demonstrated to induce anxiolytic effects in the OF test, whereas relatively low number of studies have reported that exercise triggers anxiogenic effects (Dishman et al., 1996; Grace et al., 2009). The discrepancy may relate to the combined exercise-induced processes and recovery from surgery processes, and may be a clinically relevant observation. WBV has shown protective and restorative effects on anxiety-like behavior in rats induced by the restrained stress model (Peng et al., 2021). Since in the present study, surgery did not increase anxiety-like behavior, there may not much to be gained by WBV in that regard. A significant correlation was found between early heart rate responses (i.e.: the mean heart rate values of the first 6 days) and anxiety-like behavior. The lower heart rate was correlated to a higher degree of walking distance, center visits and rearing activity, as well as to less time spent in the corners. If heart rate reflects sympathetic tone, this finding suggests that lower sympathetic tone seems to relate to lower anxiety. However, there are no significant indications that either WBV or running exercise reduce sympathetic tone in the present study (heart rate reduction). According to clinical trials the relationship between higher heart rate and/or lower heart rate variability versus intensified depressive symptoms has been reported as a relevant and important sign to analyze and detect depression (Meerwijk et al., 2014; Tessier et al., 2017).

Although we previously reported that spatial memory in MWM and NLR was impaired in the first two weeks following surgery, without affecting cognitive flexibility (Hovens et al., 2014a), different effects were observed in our current experiment. Spatial memory seems to be intact, while cognitive flexibility was declined. The latter seemed to be restored by both running exercise and WBV interventions. This discrepancy may be due to the methodical modifications. In the current study, surgery control animals underwent more intense handling and environmental stimulation due to the pseudo WBV/exercise treatments compared to our previous experiments (Hovens et al., 2014a). This may contribute to the lack of observable cognitive impairments in spatial tasks of MWM and NLR. The potential of long-term pseudo treatment (i.e.: treadmill control group) has shown to reverse age-related cognitive decline and mimic the beneficial effects of treadmill exercise group including hippocampal plasticity and expression of growth factors (O'Callaghan et al., 2009). Moreover, regular handling combined with extensive exposure to a new environment outside the home cage may be considered as an environmentally enriched condition.

Our results in the MWM could be interpreted as all animals were equally capable of spatial learning during the training trials, but the pseudo exercise - surgery animals were unable to continue when they were presented with a new (reversal) problem. Cognitive flexibility is primary associated with functionality of prefrontal cortex (PFC). However, it has also been reported that rats with a hippocampal lesion were able to acquire a place response, but failed to act correctly when they were faced with a new (reversal) problem (Whishaw and Tomie, 1997); suggesting also a role of the hippocampus in matching place problems or in series of place “reversal” problems. Active exercise is known for having beneficial influences on both PFC and hippocampal functions (van Praag, 2005; Brockett et al., 2015). However, WBV has been recently demonstrated to improve both spatial memory and hippocampal functioning in rodents (Cariati et al., 2021; Peng et al., 2021; Oroszi et al., 2022b). There are no pre-clinical data reporting on the effects of WBV in the PFC. In addition, cognitive flexibility in animal studies may reflect attention and executive functions in humans (Klanker et al., 2013) and WBV has been shown to improve attention and executive functions in patients (Fuermaier et al., 2014a, 2014b). Moreover, WBV has been considered as a feasible strategy for elderly people with high level of frailty (Heesterbeek et al., 2019). Results of another study also showed that WBV improved cognitive functions in elderly people (De Bruin; 2020). Moreover, a recent review demonstrated the effectiveness of low intensity WBV on the clinical management of dementia, and it also addressed the individual's subjective response to the intervention (Campbell et al., 2022). These instances represent the clinical aspects of WBV research on memory function and/or anxiety-like behavior. Overall, these clinical and preclinical data do not show unanimity regarding the various outcome measures. This suggests that the difference in effects of WBV on spatial memory/hippocampal functioning or cognitive flexibility could be species specific (human vs. rodents), but it may also depend on the applied WBV protocol. We speculate that adjustment of amplitude is a more crucial parameter regarding translational optimization between preclinical and clinical studies compared to adjustment of frequency or total duration of the intervention. This might be due to the different size/weight and/or body composition and/or mechanoreceptor distribution and density of the clinical and preclinical subjects.

Neither muscle strength nor motor coordination were affected by surgery, but only muscle strength was improved by active exercise intervention. This effect of physical exercise on motor performance is well known in rodents (Silveira et al., 2019; Hernández-Álvarez et al., 2019). WBV primary stimulates muscle tissue via the activation and synchronization of its motor units (Hagbarth and Eklund, 1968; Bosco et al., 1999). The MarodyneLiv system has been demonstrated to enhance neuromuscular dynamics and muscle strength in both young and old mice (Mettlach et al., 2014). In addition, we demonstrated the effectiveness of five weeks of daily single 10 min WBV intervention using the MarodyneLiv plate on the neuromuscular performance in aged rats (Oroszi et al., 2022a, b). In the present study, rats underwent more intensive WBV sessions (mostly twice a day, 7 days a week). Based on these previous observations (Mettlach et al., 2014; Oroszi et al., 2022a, 2022b), we hypothesize that either longer intervention and/or different amplitude and frequency parameters are needed to obtain beneficial effects on the musculoskeletal system. We speculate that daily 5–20 min sessions at low intensity (frequency of 30 Hz and amplitude of 50–200 μm) with the minimum total duration of 4–5 weeks of intervention is sufficient to improve muscle and osseous parameters.

Taken together, anxiogenic effects of running exercise, but not of WBV, but similar beneficial effects on cognitive performance supports the therapeutic potential of WBV in early recovery after surgery.

### 4.4 Effects on the brain

Increased microglia activation was detected in the pseudo exercise - surgery group in the CA1 region of the hippocampus, but not in the Hilus. In addition, neurogenesis (based on DCX-immunostaining) seemed to be impaired in the Dentate gyrus by surgery. Although both active (Seo et al., 2019; Mee-inta and Zhao, 2019) and passive exercises (Peng et al., 2021; Chen et al., 2022) are associated with anti-inflammatory effects, neither intervention alleviated the surgery-induced neuroinflammation. However, WBV seemed to prevent the decline in neurogenesis. Still, these effects were not accompanied with differences in spatial learning and memory.

We previously reported increased microglia activation and impaired neurogenesis in the hippocampus induced by the same type of abdominal surgery (Hovens et al., 2013, 2014a). Increased microglia activation was only detected after the first post-surgical week and was resolved from the second week onwards, whereas neurogenesis remained impaired for the first three weeks following surgery (Hovens et al., 2014a). In addition, surgical traumas and their detrimental effects in POCD including hippocampal-mediated neuroinflammation have also been demonstrated by others (Wan et al., 2007; Cibelli et al., 2010). Moreover, rather than the other areas, hippocampal CA1 microglia activity was found to be associated with post-operative cognitive dysfunction (Hovens et al., 2013), as well as with a higher degree of vulnerability to neurological diseases and age-related neurodegeneration (Michaelis, 2012). Our findings seem to be in accordance with these observations reported previously.

Ample studies have shown the meaningful therapeutic potential of physical exercise on brain functions including hippocampal plasticity and neurogenesis (van Praag, 2005; Ma et al., 2017), as well as modulation of microglia activation (Mee-inta and Zhao, 2019). Moreover, aerobic exercise has been demonstrated to ameliorate neurogenesis indicated by increased expression of BDNF and DCX positive cells in the ischemic hippocampus after stroke in rats (Luo et al., 2019; Cheng et al., 2020). Also, considerable evidence suggests that low-intensity (with a frequency of 15–40 Hz) vibration may be able to promote similar therapeutic effects as physical exercise, such as releasing neurotrophic factors (Huang et al., 2018; Raval et al., 2018) and alleviating neuroinflammation (Peng et al., 2021) in various brain regions including the hippocampus. In addition, WBV tended to have dose-response effects on synaptic plasticity in the hippocampus based on in vitro electrophysiological recording (Cariati et al., 2021). Further, WBV might increase the level of plasma BDNF and insulin-like growth factor (IGF-1) in human subjects after chronic intervention (Simão et al., 2019; Wunram; 2021).

In the current experiment, two weeks intervention with low intensity (frequency of 30 Hz, amplitude of 50–200 μm) was applied to investigate brain markers related to neuroinflammation and neurogenesis. These two weeks of WBV can be considered as a relatively short duration of intervention compared to other studies with similar brain related subjects (4–12 weeks WBV) (Huang et al., 2018; Raval et al., 2018; Cariati et al., 2021; Peng et al., 2021; Chen et al., 2022). Beneficial effects of relatively short-term (11 days) exercise intervention were demonstrated on mood and BDNF regulation (Tomiga et al., 2021). Furthermore, short-term exercise intervention (7 and 14 days) initiated two days after intracerebral hemorrhage surgery showed similar improvement on motor functional recovery (Tamakoshi et al., 2018). Nevertheless, our findings support effectiveness of a short-term WBV intervention. However, two weeks of WBV restored neurogenesis, without affecting neuroinflammation. This finding may relate to the different time courses of these parameters after surgery (Hovens et al., 2014b, Hovens et al., 2014a). Moreover, anti-inflammatory treatment with ibuprofen at the time of surgery increased neurogenesis, which was even correlated to increased microglia activity, two weeks after surgery (Oberman et al., 2021). We hypothesize that WBV might be more effective in case of significant deficits.

In addition, significant correlations were found between hippocampal neurogenesis and locomotor activity early and late after surgery, as well as between microglia activation (CA1) and locomotor activity 14 days after surgery. The higher the locomotor activity, the higher the expression of neurogenesis and the lower the level of microglia activation were observed. Literature data are limited in this context; however, the level of motor activity has been recently shown to be associated with anti-inflammatory effects (Afshari et al., 2021). We hypothesize that these beneficial effects on hippocampal neurogenesis shared by WBV and physical exercise may be mediated by similar molecular and cellular mechanisms.

## 5 Limitations

Several limitations have to be addressed. Although the design of the active exercise protocol was chosen and planned carefully, animals with telemetry transmitter surgery may experience limited hind limb functions, as a result of the partly occlusion of the abdominal aorta. This would be supported by the observation of a slightly lower distance ran over the whole protocol (Figure A1 – Supplementary materials). In addition, it has been reported previously that the size of the implanted sensor can significantly influence the voluntary running performance in mice after surgery (Helwig et al., 2012). We do not have information about this aspect in rats, but sensors were about 3.1 cc in volume and 4.4 g in weight.

Since we have used the interaction of neuroinflammation/anxiety/cognitive decline and therapeutic potential of active/passive exercise as our main hypothesis, in hindsight collecting timed blood samples to further investigate underlying mechanisms such as growth factors or proinflammatory cytokines would have been wise.

The design of the present study was based on our previous work (Hovens et al., 2014a). The experiment ended after the second post-operative week; a time point with anticipated cognitive decline, hippocampal neuroinflammation and reduced neurogenesis. This short duration focused on the effects of interventions during the early recovery from surgery, as well as to examine the ultimate behavioral consequences. In the present study, we did not observe the impaired spatial learning and memory after surgery, as we saw before (Hovens et al., 2014a), but impaired cognitive flexibility. This latter effect was previously only observed in rat models with elevated risk on POCD, such as advanced age (Hovens et al., 2013), more severe surgery (Hovens et al., 2012) or after previous inflammatory events (Hovens et al., 2015). An explanation for these differences could lie in the fact that pseudo exercise – non-surgery and pseudo exercise – surgery control animals received more intensive handling stimulation (pseudo treatment) during the current experiment compared to our previous work. This may have induced stress, which is also regarded to evoke inflammatory responses (Sturman et al., 2018).

Although the statistical analysis of data was carried out carefully, some limitations need to be emphasized. Firstly, a limitation concerns the relatively large number of used statistical tests including student T-test, one and repeated measure ANOVAs, as well as correlation analysis. Second limitation regards the fact that although the correlation analysis between the different domains of this study was planned in advance and it resulted in a total of 192 correlations. The selection and interpretation of these potential relevant correlations were subjective and thus, type 1 error inflation cannot be excluded.

Finally, since in the present study many different aspects were taken into account, including physiological peripheral parameters, different behavioral aspects, and their associated changes in the brain, in the face of interventions in early post-surgical recovery, results could provide interesting connections in this complex setting, but on the other hand makes the interpretation of the overall results challenging.

## 6 Conclusions

Aim of the study was to explore the putative therapeutic potential of WBV as alternative for active physical exercise to improve early recovery from surgery. Results demonstrated that low-intensity WBV and active running exercise share the beneficial effect of prevention of surgery-induced decline in cognitive flexibility, and recover hippocampal neurogenesis. However, in contrast to active exercise, WBV after surgery did not increase anxiety-like behavior. Although extensive evidence shows an important role for (neuro)inflammation in the development of postoperative cognitive dysfunction, effects of early interventions with exercise or WBV in the present study seemed not directly attributable to affect neuroinflammation. The potential mechanisms of action of the beneficial effects of WBV early after surgery is therefore a subject for follow-up studies.

In addition, there is increasing attention for integration of different interventional approaches prior to the surgical procedure as part of pre-habilitation process (Carli and Scheede-Bergdahl, 2015). WBV as preoperative intervention was successfully demonstrated to ameliorate neuroinflammation and glial changes after traumatic brain injury in mice (Chen et al., 2022). Hence, in addition to a post-surgical intervention, WBV may be studied as presurgical intervention as well. Our preclinical model with major abdominal surgery may provide a relevant tool. Pre and postoperative WBV and active exercise programs are effective in reducing the length of stay and number of side-complications and in improving neuromuscular performance (Fu et al., 2013; Hoogeboom et al., 2014; Hsiao et al., 2019). These findings would support that WBV, at least in part, shows similar therapeutic potentials in clinical and preclinical experiments, justifying further research on this intervention in our animal model. Further, it is important to stress out that rats were used as a model species to reveal potential underlying mechanisms of which translational values always have to be proven, however, if something works in rodents, it does not necessarily work in humans (to the same degree).

Finally, we concluded that WBV may contribute to accelerated recovery from surgery, however, it still requires further scrutiny whether WBV may provide a relevant and safe alternative for physical exercise shortly after surgery.

## Ethics statement

The animal study was reviewed and approved by the national Competent Authority (CCD) and the local ethical committee (DierExperimentenCommissie) of the university of Groningen, The Netherlands.

## Author contributions

RS, EZ, SB, BL and CN contributed to the conception and design of the study. RS and TO performed the animal experiment, the staining, organized the data and did statistical analysis; and wrote the first draft of the manuscript. RS performed part of the surgeries. KO assisted at the animal experiment. All authors contributed to the final version of the manuscript and approved the final version.

## Funding

This research received no specific funding.

## Declaration of competing interest

The authors declare that they have no known competing financial interests or personal relationships that could have appeared to influence the work reported in this paper.

## Data Availability

Data will be made available on request.
