# Supplementary material for: Whole body vibration, an alternative for exercise to improve recovery from surgery?
Source: Brain Behav Immun Health. 2022 Sep 24;26:100521. doi: 10.1016/j.bbih.2022.100521 (PMC9531049; doi:10.1016/j.bbih.2022.100521)
Supplement: Multimedia component 1 [file mmc1.docx]

**Supplementary table 1 – descriptive statistics –**

**Behavioral testing and molecular markers**

| **Behavioral Testing** | | | | | | | | |
| --- | --- | --- | --- | --- | --- | --- | --- | --- |
| **Variable** | **Groups** | **N** | **Mean** | **Median** | **Min** | **Max** | **Std.Dev** | **SEM** |
| Running Performance  AUC | Abdominal | 7 | 5262.57 | 5570.00 | 3250.00 | 5850.00 | 924.84 | 349.55 |
|  | Telemetry | 7 | 4232.57 | 4324.00 | 3470.00 | 4752.00 | 393.15 | 148.59 |
| Maximum Weight Lost | Non-Surgery Pseudo | 7 | 0.57 | 1.00 | -6.00 | 5.00 | 3.40 | 1.28 |
|  | Non-Surgery Home | 7 | -0.42 | 0.00 | -9.00 | 6.00 | 4.46 | 1.68 |
|  | Abdominal – pseudo | 8 | -16.00 | -16.00 | -22.00 | -9.00 | 4.75 | 1.67 |
|  | Telemetry - Pseudo | 8 | -26.00 | -30.50 | -38.00 | -11.00 | 10.07 | 3.56 |
|  | Abdominal - WBV | 8 | -19.87 | -20.00 | -24.00 | -15.00 | 3.18 | 1.12 |
|  | Abdominal – Running | 8 | -18.87 | -17.50 | -27.00 | -14.00 | 4.29 | 1.51 |
|  | Telemetry – WBV | 8 | -26.50 | -29.00 | -37.00 | -12.00 | 9.10 | 3.21 |
|  | Telemetry - Running | 7 | -25.42 | -29.00 | -35.00 | -11.00 | 8.71 | 3.29 |
| OF – Walking Distance | Non - Surgery | 14 | 2529.88 | 2655.50 | 1091.48 | 3853.41 | 736.16 | 196.74 |
|  | Surgery | 16 | 2079.20 | 2253.93 | 460.87 | 3224.32 | 737.02 | 184.25 |
|  | Surgery - WBV | 16 | 2142.87 | 2046.01 | 1130.67 | 3638.67 | 739.84 | 184.96 |
|  | Surgery - Running | 15 | 1473.72 | 1501.40 | 183.06 | 3443.55 | 747.11 | 192.90 |
| OF – Center Entries | Non - Surgery | 14 | 9.00 | 8.00 | 0.00 | 18.00 | 6.67 | 1.78 |
|  | Surgery | 16 | 7.87 | 7.00 | 0.00 | 19.00 | 5.84 | 1.46 |
|  | Surgery - WBV | 16 | 5.68 | 4.00 | 0.00 | 19.00 | 5.53 | 1.38 |
|  | Surgery - Running | 15 | 2.80 | 1.00 | 0.00 | 9.00 | 3.16 | 0.81 |
| OF – Time spent in the corner | Non - Surgery | 14 | 169.20 | 173.30 | 96.24 | 262.52 | 48.06 | 12.84 |
|  | Surgery | 16 | 193.70 | 200.38 | 122.52 | 274.76 | 44.80 | 11.20 |
|  | Surgery - WBV | 16 | 193.59 | 188.32 | 85.08 | 260.80 | 44.08 | 11.02 |
|  | Surgery - Running | 15 | 221.62 | 221.60 | 113.64 | 296.88 | 46.65 | 12.04 |
| OF – Rearing | Non - Surgery | 14 | 17.85 | 18.50 | 2.00 | 37.00 | 8.96 | 2.39 |
|  | Surgery | 16 | 14.25 | 13.50 | 0.00 | 29.00 | 8.85 | 2.21 |
|  | Surgery - WBV | 16 | 14.06 | 12.50 | 1.00 | 36.00 | 8.14 | 2.03 |
|  | Surgery - Running | 15 | 8.86 | 9.00 | 0.00 | 19.00 | 5.78 | 1.49 |
| NOR Preference | Non - Surgery | 14 | 58.71 | 60.67 | 0.00 | 100.00 | 27.46 | 7.34 |
|  | Surgery | 12 | 71.58 | 73.21 | 43.10 | 100.00 | 20.96 | 6.05 |
|  | Surgery - WBV | 14 | 66.89 | 68.26 | 45.07 | 92.54 | 13.98 | 3.73 |
|  | Surgery - Running | 13 | 71.31 | 70.52 | 0.00 | 100.00 | 27.22 | 7.55 |
| NLR Preference | Non - Surgery | 12 | 65.15 | 65.93 | 28.00 | 100.00 | 21.51 | 6.20 |
|  | Surgery | 13 | 68.28 | 62.66 | 44.72 | 100.00 | 15.27 | 4.23 |
|  | Surgery - WBV | 14 | 65.09 | 62.48 | 29.32 | 100.00 | 17.53 | 4.68 |
|  | Surgery - Running | 12 | 75.60 | 77.68 | 42.33 | 100.00 | 22.22 | 6.41 |
| MWM  Training | Non - Surgery | 14 | 12.30 | 10.87 | 6.15 | 21.99 | 4.40 | 1.17 |
|  | Surgery | 16 | 12.49 | 11.35 | 9.17 | 18.21 | 2.80 | 0.70 |
|  | Surgery - WBV | 16 | 14.06 | 13.27 | 7.55 | 23.65 | 4.52 | 1.13 |
|  | Surgery - Running | 15 | 12.31 | 12.25 | 7.08 | 22.84 | 3.97 | 1.02 |
| MWM  Reversal | Non - Surgery | 14 | 9.12 | 8.10 | 6.11 | 13.41 | 2.41 | 0.64 |
|  | Surgery | 16 | 9.44 | 7.48 | 4.35 | 24.64 | 5.72 | 1.43 |
|  | Surgery - WBV | 16 | 9.34 | 9.21 | 4.80 | 14.08 | 2.38 | 0.59 |
|  | Surgery - Running | 15 | 9.38 | 7.66 | 3.43 | 16.07 | 4.11 | 1.06 |
| MWM  Probe Trial 2. Platform crossing | Non - Surgery | 14 | 2.78 | 2.50 | 1.00 | 5.00 | 1.25 | 0.33 |
|  | Surgery | 16 | 3.06 | 3.00 | 0.00 | 6.00 | 1.91 | 0.47 |
|  | Surgery - WBV | 16 | 3.62 | 3.00 | 1.00 | 7.00 | 2.12 | 0.53 |
|  | Surgery - Running | 15 | 3.40 | 3.00 | 1.00 | 7.00 | 1.54 | 0.40 |
| MWM  Memory Consolidation | Non - Surgery | 14 | 7.61 | 6.88 | -1.60 | 18.15 | 5.59 | 1.49 |
|  | Surgery | 16 | 5.25 | 5.78 | -4.08 | 13.38 | 5.15 | 1.28 |
|  | Surgery - WBV | 16 | 9.79 | 10.23 | 0.80 | 20.26 | 6.47 | 1.61 |
|  | Surgery - Running | 15 | 9.77 | 7.94 | -2.97 | 24.05 | 8.79 | 2.27 |
| Grip Hanging | Non - Surgery | 13 | 18.84 | 18.48 | 11.97 | 26.21 | 4.40 | 1.22 |
|  | Surgery | 16 | 18.24 | 18.38 | 7.50 | 29.54 | 6.43 | 1.60 |
|  | Surgery - WBV | 16 | 17.51 | 17.86 | 10.08 | 28.43 | 5.46 | 1.36 |
|  | Surgery - Running | 14 | 22.68 | 22.36 | 12.59 | 36.73 | 7.15 | 1.91 |
| Balance Beam | Non - Surgery | 13 | 6.53 | 6.38 | 3.72 | 11.42 | 2.20 | 0.61 |
|  | Surgery | 16 | 6.24 | 5.36 | 3.19 | 11.67 | 2.68 | 0.67 |
|  | Surgery - WBV | 16 | 6.19 | 6.00 | 2.71 | 10.93 | 2.44 | 0.61 |
|  | Surgery - Running | 10 | 6.48 | 6.60 | 2.33 | 10.66 | 2.82 | 0.89 |
| **Microglia Activation and DCX+** | | | | | | | | |
| **Variable** | **Groups** | **N** | **Mean** | **Median** | **Min** | **Max** | **Std.Dev** | **SEM** |
| Microglia activation – CA1 | Non - Surgery | 10 | 4.01 | 3.79 | 2.91 | 5.34 | 0.82 | 0.26 |
|  | Surgery | 13 | 5.69 | 5.80 | 3.25 | 8.27 | 1.60 | 0.44 |
|  | Surgery - WBV | 14 | 4.71 | 4.29 | 2.90 | 7.73 | 1.56 | 0.41 |
|  | Surgery - Running | 11 | 4.66 | 4.21 | 3.32 | 6.47 | 0.98 | 0.29 |
| Microglia Activation - Hilus | Non - Surgery | 9 | 13.33 | 12.68 | 4.97 | 19.19 | 4.04 | 1.35 |
|  | Surgery | 13 | 14.92 | 14.96 | 9.02 | 21.12 | 3.46 | 0.96 |
|  | Surgery - WBV | 15 | 4.35 | 4.11 | 2.77 | 6.86 | 0.99 | 0.25 |
|  | Surgery - Running | 12 | 14.37 | 14.79 | 8.84 | 20.62 | 3.71 | 1.07 |
| Microglia Coverage – CA1 | Non - Surgery | 10 | 0.10 | 0.10 | 0.06 | 0.16 | 0.03 | 0.01 |
|  | Surgery | 14 | 0.14 | 0.14 | 0.11 | 0.18 | 0.02 | 0.01 |
|  | Surgery - WBV | 14 | 0.14 | 0.14 | 0.10 | 0.18 | 0.02 | 0.01 |
|  | Surgery - Running | 11 | 0.13 | 0.13 | 0.09 | 0.16 | 0.02 | 0.01 |
| Microglia Coverage – Hilus | Non - Surgery | 10 | 0.15 | 0.14 | 0.11 | 0.19 | 0.02 | 0.01 |
|  | Surgery | 14 | 0.10 | 0.10 | 0.06 | 0.15 | 0.02 | 0.01 |
|  | Surgery - WBV | 15 | 0.12 | 0.12 | 0.09 | 0.14 | 0.01 | 0.00 |
|  | Surgery - Running | 12 | 0.11 | 0.11 | 0.08 | 0.13 | 0.02 | 0.00 |
| Microglia Cell Body – CA1 | Non - Surgery | 10 | 439.07 | 434.54 | 371.69 | 493.96 | 43.36 | 13.71 |
|  | Surgery | 14 | 471.33 | 451.18 | 337.38 | 652.63 | 89.11 | 23.82 |
|  | Surgery - WBV | 15 | 440.58 | 437.36 | 338.25 | 606.50 | 71.55 | 18.47 |
|  | Surgery - Running | 12 | 447.39 | 443.03 | 338.46 | 591.00 | 67.54 | 19.50 |
| Microglia Cell Body – Hilus | Non - Surgery | 10 | 404.16 | 393.31 | 350.91 | 536.80 | 54.24 | 17.15 |
|  | Surgery | 14 | 375.07 | 390.74 | 285.14 | 450.59 | 48.99 | 13.09 |
|  | Surgery - WBV | 15 | 576.98 | 379.95 | 315.55 | 2921.48 | 653.52 | 168.74 |
|  | Surgery - Running | 12 | 379.50 | 370.07 | 299.17 | 457.86 | 46.42 | 13.40 |
| Microglia Dendrites – CA1 | Non - Surgery | 10 | 11160.23 | 11939.85 | 7172.21 | 13888.08 | 2165.44 | 684.77 |
|  | Surgery | 13 | 8897.69 | 8413.69 | 5533.68 | 14322.90 | 2644.06 | 733.33 |
|  | Surgery - WBV | 14 | 8969.86 | 8853.67 | 4921.68 | 14334.20 | 2385.27 | 637.49 |
|  | Surgery - Running | 12 | 9296.07 | 8662.23 | 5721.02 | 12542.93 | 2051.72 | 592.28 |
| Microglia Dendrites - Hilus | Non - Surgery | 9 | 2531.18 | 2733.10 | 1028.31 | 4102.18 | 916.76 | 305.59 |
|  | Surgery | 14 | 2289.87 | 2440.08 | 1216.59 | 3259.69 | 662.46 | 177.05 |
|  | Surgery - WBV | 15 | 2876.15 | 2539.76 | 1746.89 | 4811.31 | 968.72 | 250.12 |
|  | Surgery - Running | 12 | 2569.34 | 2424.74 | 1533.30 | 4663.18 | 837.95 | 241.90 |
| Microglia Total Cell Size CA1 | Non - Surgery | 10 | 11599.30 | 12385.28 | 7564.50 | 14382.04 | 2172.04 | 686.86 |
|  | Surgery | 13 | 9379.32 | 8987.29 | 5965.61 | 14794.58 | 2653.09 | 735.84 |
|  | Surgery - WBV | 14 | 9415.26 | 9241.36 | 5426.70 | 14771.57 | 2347.13 | 627.30 |
|  | Surgery - Running | 12 | 9743.46 | 9118.15 | 6185.44 | 12973.73 | 2056.39 | 593.63 |
| Microglia Total Cell Size - Hilus | Non - Surgery | 9 | 2933.60 | 3120.20 | 1394.90 | 4528.97 | 936.48 | 312.16 |
|  | Surgery | 14 | 2664.94 | 2819.28 | 1612.14 | 3657.47 | 672.96 | 179.86 |
|  | Surgery - WBV | 15 | 3283.81 | 2921.48 | 2110.42 | 5307.69 | 1012.59 | 261.45 |
|  | Surgery - Running | 11 | 2751.89 | 2784.84 | 1931.70 | 3927.06 | 542.08 | 163.44 |
| Microglia per AOI CA1 | Non - Surgery | 10 | 0.000013 | 0.000014 | 0.000011 | 0.000016 | 0.000002 | 0.000001 |
|  | Surgery | 13 | 0.000015 | 0.000011 | 0.000021 | 0.000003 | 0.000013 | 0.000015 |
|  | Surgery - WBV | 15 | 0.000015 | 0.000015 | 0.000011 | 0.000020 | 0.000003 | 0.000001 |
|  | Surgery - Running | 12 | 0.000014 | 0.000014 | 0.000012 | 0.000016 | 0.000001 | 0.000000 |
| Microglia per AOI - Hilus | Non - Surgery | 10 | 0.000034 | 0.000031 | 0.000019 | 0.000050 | 0.000010 | 0.000003 |
|  | Surgery | 14 | 0.000039 | 0.000038 | 0.000023 | 0.000057 | 0.000010 | 0.000003 |
|  | Surgery - WBV | 15 | 0.000038 | 0.000036 | 0.000021 | 0.000057 | 0.000010 | 0.000003 |
|  | Surgery - Running | 12 | 0.000040 | 0.000041 | 0.000024 | 0.000052 | 0.000008 | 0.000002 |
| DCX+ (area/length) | Non - Surgery | 13 | 4.09 | 4.24 | 2.76 | 5.22 | 0.70 | 0.19 |
|  | Surgery | 15 | 3.55 | 3.58 | 2.28 | 4.34 | 0.54 | 0.14 |
|  | Surgery - WBV | 15 | 4.29 | 4.29 | 2.83 | 5.71 | 0.82 | 0.21 |
|  | Surgery - Running | 15 | 4.07 | 4.13 | 2.17 | 6.04 | 1.02 | 0.26 |

**Supplementary Table 2. Descriptive statistics – Telemetry measures**

| **Telemetry Long-Term Effects** | | | | | | | | | |
| --- | --- | --- | --- | --- | --- | --- | --- | --- | --- |
|  | **Variable** | **Groups** | **N** | **Mean** | **Median** | **Min** | **Max** | **Std.Dev** | **SEM** |
| Heart Rate | **Mean** | Surgery | 6 | 344.20 | 337.87 | 329.57 | 364.63 | 13.81 | 5.64 |
|  |  | Surgery - WBV | 8 | 351.21 | 352.93 | 310.24 | 377.85 | 21.11 | 7.46 |
|  |  | Surgery - Running | 6 | 345.65 | 347.65 | 326.58 | 363.50 | 13.80 | 5.63 |
|  | Circadian | Surgery | 6 | 43.11 | 42.10 | 30.62 | 59.48 | 10.96 | 4.47 |
|  |  | Surgery - WBV | 8 | 25.04 | 32.79 | -15.59 | 52.00 | 22.47 | 7.94 |
|  |  | Surgery - Running | 6 | 34.14 | 31.56 | 26.01 | 45.77 | 7.62 | 3.11 |
| Temperature | Mean | Surgery | 6 | 37.61 | 37.60 | 37.53 | 37.69 | 0.07 | 0.03 |
|  |  | Surgery - WBV | 8 | 37.68 | 37.67 | 37.54 | 37.83 | 0.09 | 0.03 |
|  |  | Surgery - Running | 7 | 37.58 | 37.59 | 37.40 | 37.71 | 0.11 | 0.04 |
|  | Circadian | Surgery | 6 | 0.62 | 0.72 | 0.12 | 0.97 | 0.33 | 0.13 |
|  |  | Surgery - WBV | 8 | 0.36 | 0.56 | -0.39 | 0.86 | 0.46 | 0.16 |
|  |  | Surgery - Running | 7 | 0.56 | 0.61 | 0.35 | 0.65 | 0.11 | 0.04 |
| Blood Pressure | Mean | Surgery | 4 | 107.56 | 109.52 | 98.37 | 112.84 | 6.65 | 3.33 |
|  |  | Surgery - WBV | 7 | 114.33 | 117.09 | 99.05 | 122.32 | 7.96 | 3.01 |
|  |  | Surgery - Running | 3 | 110.40 | 111.02 | 100.72 | 119.45 | 9.38 | 5.42 |
|  | Circadian | Surgery | 4 | 0.71 | 0.19 | -0.75 | 3.21 | 1.80 | 0.90 |
|  |  | Surgery - WBV | 7 | 1.39 | 0.48 | -1.36 | 5.44 | 2.50 | 0.95 |
|  |  | Surgery - Running | 3 | 0.39 | 0.50 | -1.02 | 1.69 | 1.36 | 0.78 |
| Activity | **Mean** | Surgery | 7 | 2.27 | 2.27 | 1.81 | 2.77 | 0.31 | 0.12 |
|  |  | Surgery - WBV | 8 | 2.61 | 2.75 | 1.88 | 2.92 | 0.36 | 0.13 |
|  |  | Surgery - Running | 7 | 2.25 | 2.12 | 1.62 | 3.09 | 0.52 | 0.20 |
|  | Circadian | Surgery | 7 | 1.79 | 1.68 | 0.71 | 2.80 | 0.68 | 0.26 |
|  |  | Surgery - WBV | 8 | 1.51 | 1.87 | -0.11 | 3.12 | 1.14 | 0.40 |
|  |  | Surgery - Running | 7 | 1.40 | 1.36 | 1.02 | 1.66 | 0.22 | 0.08 |
| **Telemetry Short-Term Effects** | | | | | | | | | |
|  | **Variable** | **Groups** | **N** | **Mean** | **Median** | **Min** | **Max** | **Std.Dev** | **SEM** |
| Heart  Rate | Mean | Surgery | 6 | 354.94 | 349.60 | 341.70 | 374.25 | 13.11 | 5.35 |
|  |  | Surgery - WBV | 8 | 350.51 | 351.16 | 333.31 | 378.82 | 15.83 | 5.60 |
|  |  | Surgery - Running | 6 | 349.02 | 352.69 | 326.39 | 363.08 | 13.76 | 5.62 |
|  | Circadian | Surgery | 6 | 29.09 | 28.86 | 19.20 | 39.73 | 7.44 | 3.04 |
|  |  | Surgery - WBV | 8 | 28.29 | 28.57 | 17.99 | 37.48 | 6.04 | 2.14 |
|  |  | Surgery - Running | 6 | 33.77 | 34.72 | 23.28 | 43.99 | 7.59 | 3.10 |
| Temperature | Mean | Surgery | 7 | 37.87 | 37.83 | 37.77 | 38.01 | 0.10 | 0.04 |
|  |  | Surgery - WBV | 8 | 37.82 | 37.81 | 37.72 | 37.92 | 0.06 | 0.02 |
|  |  | Surgery - Running | 7 | 37.81 | 37.80 | 37.63 | 37.99 | 0.11 | 0.04 |
|  | Circadian | Surgery | 7 | 0.49 | 0.49 | 0.30 | 0.59 | 0.10 | 0.04 |
|  |  | Surgery - WBV | 8 | 0.47 | 0.49 | 0.25 | 0.72 | 0.16 | 0.06 |
|  |  | Surgery - Running | 7 | 0.43 | 0.44 | 0.35 | 0.49 | 0.05 | 0.02 |
| Blood  Pressure | Mean | Surgery | 5 | 113.05 | 113.48 | 98.95 | 123.09 | 8.91 | 3.98 |
|  |  | Surgery - WBV | 7 | 114.45 | 115.10 | 94.83 | 124.97 | 9.86 | 3.73 |
|  |  | Surgery - Running | 5 | 109.40 | 113.31 | 94.46 | 121.30 | 12.62 | 5.64 |
|  | Circadian | Surgery | 5 | 1.78 | 1.60 | 0.33 | 2.89 | 1.03 | 0.46 |
|  |  | Surgery - WBV | 7 | 1.20 | 0.86 | -0.40 | 4.13 | 1.65 | 0.63 |
|  |  | Surgery - Running | 5 | 1.18 | -0.07 | -0.65 | 6.90 | 3.21 | 1.44 |
| Acitivity | Mean | Surgery | 7 | 1.71 | 1.60 | 1.40 | 2.10 | 0.27 | 0.10 |
|  |  | Surgery - WBV | 8 | 2.15 | 2.09 | 1.59 | 2.79 | 0.39 | 0.14 |
|  |  | Surgery - Running | 7 | 2.05 | 1.73 | 1.42 | 3.04 | 0.68 | 0.26 |
|  | Circadian | Surgery | 7 | 0.96 | 1.00 | 0.47 | 1.37 | 0.31 | 0.12 |
|  |  | Surgery - WBV | 8 | 1.41 | 1.41 | 0.69 | 2.01 | 0.47 | 0.16 |
|  |  | Surgery - Running | 7 | 1.46 | 1.25 | 0.81 | 3.37 | 0.88 | 0.33 |

**Supplementary Table 3. - One-Way ANOVA**

| **Variable** | **F value** | **p** | **Partial eta-squared** | **Observed Power** | **Post Hoc. (Tukey HSD)** |
| --- | --- | --- | --- | --- | --- |
| OF – Walking Distance | F(3.57) = 5.08 | 0.003 | 0.21 | 0.90 | Run vs. WBV = 0.06  Run vs. Surgery = 0.11  Run vs. Non-Surgery = 0.00  WBV vs. Surgery = 0.99  WBV vs. Non-Surgery = 0.48  Surgery vs. Non-Surgery = 0.35 |
| OF – Center Entries | F(3.57) = 3.72 | 0.016 | 0.16 | 0.78 | Run vs. WBV = 0.45  Run vs. Surgery = 0.05  Run vs. Non-Surgery = 0.01  WBV vs. Surgery = 0.66  WBV vs. Non-Surgery = 0.35  Surgery vs. Non-Surgery = 0.94 |
| OF – Time spent in the corner | F(3.57) = 3.17 | 0.03 | 0.14 | 0.70 | Run vs. WBV = 0.33  Run vs. Surgery = 0.33  Run vs. Non-Surgery = 0.01  WBV vs. Surgery = 1.00  WBV vs. Non-Surgery = 0.47  Surgery vs. Non-Surgery = 0.46 |
| OF – Rearing | F(3.57) = 3.09 | 0.03 | 0.13 | 0.69 | Run vs. WBV = 0.28  Run vs. Surgery = 0.25  Run vs. Non-Surgery = 0.01  WBV vs. Surgery = 0.99  WBV vs. Non-Surgery = 0.57  Surgery vs. Non-Surgery = 0.61 |
| NOR Preference | F(3.49) = 0.91 | 0.44 | 0.05 | 0.23 |  |
| SOR  Preference | F(3.47) = 0.81 | 0.49 | 0.04 | 0.21 |  |
| Probe Trial 2. Platform crossing | F(3.57) = 0.66 | 0.57 | 0.03 | 0.18 |  |
| Memory Consolidation | F(3.57) = 1.66 | 0.18 | 0.08 | 0.41 |  |
| Grip Hanging | F(3.55)  2.15 | 0.10 | 0.10 | 0.51 | Run vs. WBV = 0.09  Run vs. Surgery = 0.19  Run vs. Non-Surgery = 0.35  WBV vs. Surgery = 0.98  WBV vs. Non-Surgery = 0.93  Surgery vs. Non-Surgery = 0.99 |
| Balance Beam | F(3.51)=  0.06 | 0.98 | 0.00 | 0.06 |  |
| **Variable** | **F value** | **p** | **Partial eta-squared** | **Observed Power** | **Post Hoc. (Tukey HSD)** |
| Microglia activation – CA1 | F(3.44)=  3.13 | 0.03 | 0.17 | 0.68 | Run vs. WBV = 0.99  Run vs. Surgery = 0.25  Run vs. Non-Surgery = 0.68  WBV vs. Surgery = 0.24  WBV vs. Non-Surgery = 0.59  Surgery vs. Non-Surgery = 0.02 |
| Microglia Activation - Hilus | F(3.45)=  0.44 | 0.72 | 0.02 | 0.13 |  |
| Microglia Coverage – CA1 | F(3.45)=  1.23 | 0.30 | 0.07 | 0.30 |  |
| Microglia Coverage – Hilus | F(3.47)=  1.81 | 0.15 | 0.10 | 0.44 |  |
| Microglia Cell Body – CA1 | F(3.47)=  0.58 | 0.63 | 0.03 | 0.16 |  |
| Microglia Cell Body – Hilus | F(3.47)=  1.02 | 0.38 | 0.06 | 0.26 |  |
| Microglia Dendrites – CA1 | F(3.45)=  2.23 | 0.09 | 0.12 | 0.52 | Run vs. WBV = 0.98  Run vs. Surgery = 0.97  Run vs. Non-Surgery = 0.25  WBV vs. Surgery = 0.99  WBV vs. Non-Surgery = 0.12  Surgery vs. Non-Surgery = 0.11 |
| Microglia Dendrites - Hilus | F(3.46)=  1.15 | 0.33 | 0.07 | 0.29 |  |
| Microglia Total Cell Size CA1 | F(3.45)=  2.19 | 0.10 | 0.12 | 0.52 | Run vs. WBV = 0.98  Run vs. Surgery = 0.97  Run vs. Non-Surgery = 0.26  WBV vs. Surgery = 0.99  WBV vs. Non-Surgery = 0.12  Surgery vs. Non-Surgery = 0.12 |
| Microglia Total Cell Size - Hilus | F(3.45)=  1.59 | 0.20 | 0.09 | 0.39 |  |
| Microglia per AOI CA1 | F(3.46)=  2.08 | 0.11 | 0.11 | 0.49 |  |
| Microglia per AOI - Hilus | F(3.47)=  0.84 | 0.47 | 0.05 | 0.21 |  |
| DCX+ (area/length) | F(3.54)=  2.35 | 0.08 | 0.11 | 0.56 | Run vs. WBV = 0.86  Run vs. Surgery = 0.29  Run vs. Non-Surgery = 0.99  WBV vs. Surgery = 0.06  WBV vs. Non-Surgery = 0.91  Surgery vs. Non-Surgery = 0.28 |
| **Variable** | | **F** | **P** | **Partial eta-squared** | **Observed Power** |
| Heart Rate | **Mean** | F(2.17)=  0.33 | 0.72 | 0.03 | 0.09 |
|  | **Circadian** | F(2.17)=  2.16 | 0.14 | 0.20 | 0.38 |
| Blood Pressure | **Mean** | F(2.11)=  0.97 | 0.40 | 0.15 | 0.17 |
|  | **Circadian** | F(2.11)=  0.26 | 0.76 | 0.04 | 0.08 |
| Temperature | **Mean** | F(2.18)=  2.44 | 0.11 | 0.21 | 0.42 |
|  | **Circadian** | F(2.18)=  1.13 | 0.34 | 0.11 | 0.21 |
| Activity | **Mean** | F(2.19)=  1.93 | 0.17 | 0.16 | 0.35 |
|  | **Circadian** | F(2.19)=  0.43 | 0.65 | 0.04 | 0.11 |

**Supplementary Table 4. – Morris Water Maze – Repeated Measure ANOVA**

| **Morris Water Maze** | | | | | | |
| --- | --- | --- | --- | --- | --- | --- |
| **Variable** | **F value** | | **p** | **Partial eta-squared** | **Observed Power** |  |
| **Training** | **Group**  F(3.57) = 0.72 | | 0.54 | 0.03 | 0.19 |  |
|  | **Trial**  F(5.285) = 142.97 | | 0.00 | 0.71 | 1.00 |  |
|  | **Trial*group**  F(15.285) = 1.30 | | 0.20 | 0.06 | 0.79 |  |
| **Reversal** | **Group**  F(3.57) = 0.01 | | 0.99 | 0.00 | 0.05 |  |
|  | **Trial**  F(2.11) = 37.70 | | 0.00 | 0.39 | 1.00 |  |
|  | **Trial*group**  F(6.114) = 2.73 | | 0.01 | 0.12 | 0.85 | Running: 0.001 – 0.0001  WBV: 0.001 – 0.0008  Surgery: 1.000 – 0.449  Non-Surgery: 0.05 – 0.0006 |
| **Telemetry Measures – Short-term** | | | | | | |
| **Variable** | **F value** | | **p** | **Partial eta-squared** | **Observed Power** |  |
| Heart Rate | **Absolute**  **(dark + light phase)** | **Group:**  F(2.27) = 0.36 | 0.69 | 0.02 | 0.10 |  |
|  |  | **Dark/Light**  F(1.27) = 33.79 | 0.00 | 0.55 | 0.99 |  |
|  |  | **Group x Dark/Light:**  F(2.27) = 0.11 | 0.88 | 0.00 | 0.06 |  |
|  |  | **Day:**  F(5.13) = 11.45 | 0.00 | 0.29 | 0.99 |  |
|  |  | **Day x Group:**  F(10.13) = 1.00 | 0.43 | 0.06 | 0.51 |  |
|  |  | **Day x Dark/Light**  F(5.13) = 7.10 | 0.00 | 0.20 | 0.99 |  |
|  |  | **Day x Group x Dark/Light**  F(10.13) = 0.42 | 0.93 | 0.03 | 0.21 |  |
|  | **Circadian**  **(delta dark – light phase)** | **Group:**  F(2.12) = 0.84 | 0.45 | 0.12 | 0.16 |  |
|  |  | **Day:**  F(5.60) = 7.98 | 0.00 | 0.40 | 1.00 |  |
|  |  | **Day x Group:**  F(10.60) = 0.76 | 0.66 | 0.11 | 0.36 |  |
| Temperature | **Absolute**  **(dark + light phase)** | **Group:**  F(2.34) = 1.14 | 0.33 | 0.06 | 0.23 |  |
|  |  | **Dark/Light**  F(1.34) = 196.5 | 0.00 | 0.85 | 1.00 |  |
|  |  | **Group x Dark/Light:**  F(2.34) = 0.81 | 0.44 | 0.04 | 0.17 |  |
|  |  | **Day:**  F(5.17) = 27.08 | 0.00 | 0.44 | 1.00 |  |
|  |  | **Day x Group:**  F(10.17) = 1.78 | 0.06 | 0.09 | 0.82 |  |
|  |  | **Day x Dark/Light**  F(5.17) = 10.46 | 0.00 | 0.23 | 0.99 |  |
|  |  | **Day x Group x Dark/Light**  F(10.17) = 0.41 | 0.93 | 0.02 | 0.21 |  |
|  | **Circadian** | **Group:**  F(2.16) = 0.51 | 0.61 | 0.06 | 0.12 |  |
|  |  | **Day:**  F (5.80) = 22.04 | 0.00 | 0.58 | 1.00 |  |
|  |  | **Day x Group:**  F(10.80) = 0.69 | 0.72 | 0.08 | 0.34 |  |
| Blood Pressure | **Absolute**  **(dark + light phase** | **Group:**  F(2.23) = 0.64 | 0.53 | 0.05 | 0.14 |  |
|  |  | **Dark/Light**  F(1.23) = 0.45 | 0.50 | 0.01 | 0.09 |  |
|  |  | **Group x Dark/Light:**  F(2.23) = 0.01 | 0.98 | 0.00 | 0.05 |  |
|  |  | **Day:**  F(5.11) = 4.19 | 0.00 | 0.15 | 0.95 |  |
|  |  | **Day x Group:**  F(10.11) = 1.15 | 0.33 | 0.09 | 0.57 |  |
|  |  | **Day x Dark/Light**  F(5.11) = 2.12 | 0.06 | 0.08 | 0.68 |  |
|  |  | **Day x Group x Dark/Light**  F(10.11) = 0.29 | 0.98 | 0.02 | 0.15 |  |
|  | **Circadian** | **Group:**  F(2.10) = 2.89 | 0.10 | 0.37 | 0.44 |  |
|  |  | **Day:**  F(5.50) = 5.07 | 0.00 | 0.34 | 0.97 |  |
|  |  | **Day x Group:**  F(10.50) = 1.29 | 0.26 | 0.21 | 0.59 |  |
| Activity | **Absolute**  **(dark + light phase** | **Group:**  F(2.34) = 2.05 | 0.14 | 0.10 | 0.39 |  |
|  |  | **Dark/Light**  F(1.34) = 49.95 | 0.00 | 0.59 | 1.00 |  |
|  |  | **Group x Dark/Light:**  F(2.34) = 0.71 | 0.49 | 0.04 | 0.16 |  |
|  |  | **Day:**  F(5.17) = 4.30 | 0.00 | 0.11 | 0.96 |  |
|  |  | **Day x Group:**  F(10.17) = 1.59 | 0.11 | 0.08 | 0.76 |  |
|  |  | **Day x Dark/Light**  F(5.17) = 4.58 | 0.00 | 0.11 | 0.97 |  |
|  |  | **Day x Group x Dark/Light**  F(10.17) = 0.82 | 0.60 | 0.04 | 0.42 |  |
|  | **Circadian** | **Group:**  F(2.17) = 1.28 | 0.30 | 0.13 | 0.24 |  |
|  |  | **Day:**  F(5.85) = 4.71 | 0.00 | 0.22 | 0.97 |  |
|  |  | **Day x Group:**  F(10.85) = 1.05 | 0.41 | 0.11 | 0.52 |  |

**Additional figures and tables**

**Figure 1. Running Performance.** Differences in avarage running distance (meter/30minutes) during the 15 days of the intervention (Panel A). Commulative running distance (AUC) was significantly increased in the abdominal surgery – running group compared to telemetry surgery – running group (Panel B). Data are depicted as mean ± SEM. *: P < .05.

**Figure 2. Spatial and novel object recognition memory.** Effects of surgery (pseudo exercise), vibration and running interventions on the preference time in novel object relocation and novel object recognition tests. Significant alteration was not observed in these parameters. Data are depicted as mean and standard error of mean. (NLR - NOR: non – surgery n = 12 - 13, surgery n = 13 - 12, surgery + WBV n = 14 - 14, surgery + running n = 12 - 14)

**Figure 3. Microglia Activation.** Effects of surgery (pseudo exercise, vibration and running interventions on microglia activation in the prefrontal cortex. Significant alteration was not observed between the experimental groups. Data are depicted as mean and standard error of mean.

|  |  | **Open Field** | | | | **NLR Preference** | **NOR Preference** | **Motor Performance** | | **Microglia Activation** | | | **Neurogenesis** |
| --- | --- | --- | --- | --- | --- | --- | --- | --- | --- | --- | --- | --- | --- |
|  |  | **Distance Moved** | **Center Entries** | **Corner Time** | **Rearing** |  |  | **Grip Hanging** | **Walking Time** | **CA1 Activation** | **Hilus Activation** | **PFC Activation** | **DCX+** |
| **Heart Rate** | **Mean Day 1 -Day 6** | -0.27 | -0.50 | 0.42 | -0.51 | 0.31 | 0.01 | 0.24 | -0.23 | -0.33 | -0.21 | -0.21 | 0.20 |
|  |  | p=.242 | p=.023 | p=.066 | p=.021 | p=.259 | p=.981 | p=.301 | p=.372 | p=.195 | p=.400 | p=.398 | p=.408 |
|  | **Mean Day 14** | -0.16 | -0.31 | 0.29 | -0.39 | 0.19 | -0.22 | 0.38 | -0.46 | -0.63 | -0.30 | 0.04 | 0.31 |
|  |  | p=.488 | p=.185 | p=.219 | p=.087 | p=.499 | p=.403 | p=.100 | p=.061 | p=.006 | p=.230 | p=.873 | p=.197 |
|  | **Day 1 - Day 6 Circadian** | 0.32 | 0.03 | 0.04 | 0.08 | 0.14 | 0.22 | 0.35 | -0.20 | -0.16 | -0.05 | 0.02 | -0.04 |
|  |  | p=.174 | p=.892 | p=.853 | p=.734 | p=.609 | p=.390 | p=.131 | p=.436 | p=.550 | p=.830 | p=.925 | p=.871 |
|  | **Day 14 Cricadian** | 0.28 | 0.34 | -0.25 | 0.08 | 0.20 | 0.24 | 0.21 | 0.32 | -0.02 | -0.06 | 0.07 | 0.07 |
|  |  | p=.227 | p=.149 | p=.288 | p=.729 | p=.475 | p=.361 | p=.368 | p=.216 | p=.931 | p=.799 | p=.766 | p=.787 |
| **Temperature** | **Mean Day 1 -Day 6** | -0.20 | 0.20 | 0.19 | -0.27 | 0.14 | -0.12 | 0.16 | 0.25 | -0.12 | -0.04 | -0.05 | -0.30 |
|  |  | p=.366 | p=.374 | p=.401 | p=.229 | p=.595 | p=.649 | p=.464 | p=.302 | p=.637 | p=.868 | p=.845 | p=.186 |
|  | **Mean Day 14** | -0.14 | 0.08 | 0.21 | -0.29 | 0.00 | -0.20 | -0.02 | -0.09 | -0.16 | 0.13 | 0.18 | -0.37 |
|  |  | p=.537 | p=.734 | p=.365 | p=.206 | p=.989 | p=.432 | p=.923 | p=.730 | p=.540 | p=.607 | p=.468 | p=.106 |
|  | **Day 1 - Day 6 Circadian** | 0.44 | 0.38 | -0.46 | 0.27 | 0.08 | 0.05 | -0.10 | 0.21 | -0.07 | -0.02 | 0.18 | -0.15 |
|  |  | p=.043 | p=.083 | p=.031 | p=.217 | p=.765 | p=.858 | p=.654 | p=.379 | p=.770 | p=.936 | p=.445 | p=.504 |
|  | **Day 14 Cricadian** | 0.07 | 0.23 | -0.16 | 0.01 | 0.32 | 0.02 | 0.15 | 0.12 | -0.22 | -0.13 | 0.16 | -0.02 |
|  |  | p=.772 | p=.310 | p=.501 | p=.962 | p=.221 | p=.934 | p=.509 | p=.635 | p=.387 | p=.617 | p=.521 | p=.931 |
| **Blood Pressure** | **Mean Day 1 -Day 6** | 0.37 | 0.16 | -0.35 | 0.37 | -0.08 | -0.39 | -0.51 | 0.31 | 0.37 | 0.10 | -0.02 | -0.12 |
|  |  | p=.145 | p=.538 | p=.168 | p=.146 | p=.784 | p=.147 | p=.036 | p=.264 | p=.189 | p=.710 | p=.939 | p=.652 |
|  | **Mean Day 14** | 0.19 | 0.03 | -0.33 | 0.16 | 0.32 | -0.43 | -0.57 | 0.29 | 0.35 | 0.00 | 0.05 | -0.30 |
|  |  | p=.526 | p=.930 | p=.251 | p=.585 | p=.362 | p=.145 | p=.033 | p=.335 | p=.258 | p=1.00 | p=.853 | p=.294 |
|  | **Day 1 - Day 6 Circadian** | -0.11 | -0.06 | 0.13 | -0.28 | -0.25 | 0.08 | 0.20 | 0.21 | -0.25 | -0.25 | -0.42 | 0.31 |
|  |  | p=.672 | p=.810 | p=.619 | p=.269 | p=.411 | p=.773 | p=.439 | p=.442 | p=.395 | p=.360 | p=.107 | p=.230 |
|  | **Day 14 Cricadian** | -0.21 | -0.32 | 0.08 | -0.35 | -0.08 | -0.22 | -0.11 | -0.09 | -0.35 | -0.33 | -0.48 | 0.47 |
|  |  | p=.470 | p=.267 | p=.779 | p=.227 | p=.831 | p=.464 | p=.697 | p=.759 | p=.272 | p=.273 | p=.083 | p=.088 |
| **Activity** | **Mean Day 1 -Day 6** | 0.03 | -0.35 | 0.26 | 0.01 | 0.03 | -0.19 | -0.10 | -0.37 | -0.18 | -0.18 | -0.18 | 0.57 |
|  |  | p=.901 | p=.114 | p=.248 | p=.979 | p=.920 | p=.452 | p=.654 | p=.123 | p=.467 | p=.458 | p=.458 | p=.007 |
|  | **Mean Day 14** | -0.02 | -0.28 | 0.21 | -0.01 | -0.18 | -0.59 | 0.03 | -0.46 | -0.50 | -0.21 | -0.17 | 0.67 |
|  |  | p=.930 | p=.205 | p=.344 | p=.972 | p=.513 | p=.009 | p=.902 | p=.045 | p=.034 | p=.394 | p=.462 | p=.001 |
|  | **Day 1 - Day 6 Circadian** | 0.07 | -0.32 | 0.17 | 0.08 | 0.17 | -0.23 | -0.13 | -0.30 | -0.35 | -0.33 | -0.24 | 0.36 |
|  |  | p=.743 | p=.141 | p=.459 | p=.732 | p=.525 | p=.357 | p=.562 | p=.210 | p=.152 | p=.173 | p=.312 | p=.106 |
|  | **Day 14 Cricadian** | -0.06 | -0.05 | 0.01 | -0.22 | 0.33 | -0.05 | 0.26 | -0.05 | -0.51 | -0.15 | 0.30 | 0.11 |
|  |  | p=.803 | p=.820 | p=.956 | p=.316 | p=.214 | p=.837 | p=.238 | p=.828 | p=.031 | p=.547 | p=.199 | p=.635 |
